# Supplementary material for: Comprehensive Molecular Analysis Identified an SRSF Family-Based Score for Prognosis and Therapy Efficiency Prediction in Hepatocellular Carcinoma
Source: Cancers (Basel). 2022 Sep 28;14(19):4727. doi: 10.3390/cancers14194727 (PMC9563307; doi:10.3390/cancers14194727)
Supplement: Supplementary file 1 [file cancers-14-04727-s001.zip › cancers-1910144-supplementary.pdf]

cancers

MDPI

Supplementary Materials

Comprehensive Molecular Analysis Identified an SRSF Family-Based Score for Prognosis and Therapy Efficiency Prediction in Hepatocellular Carcinoma

1. Supplementary Figures

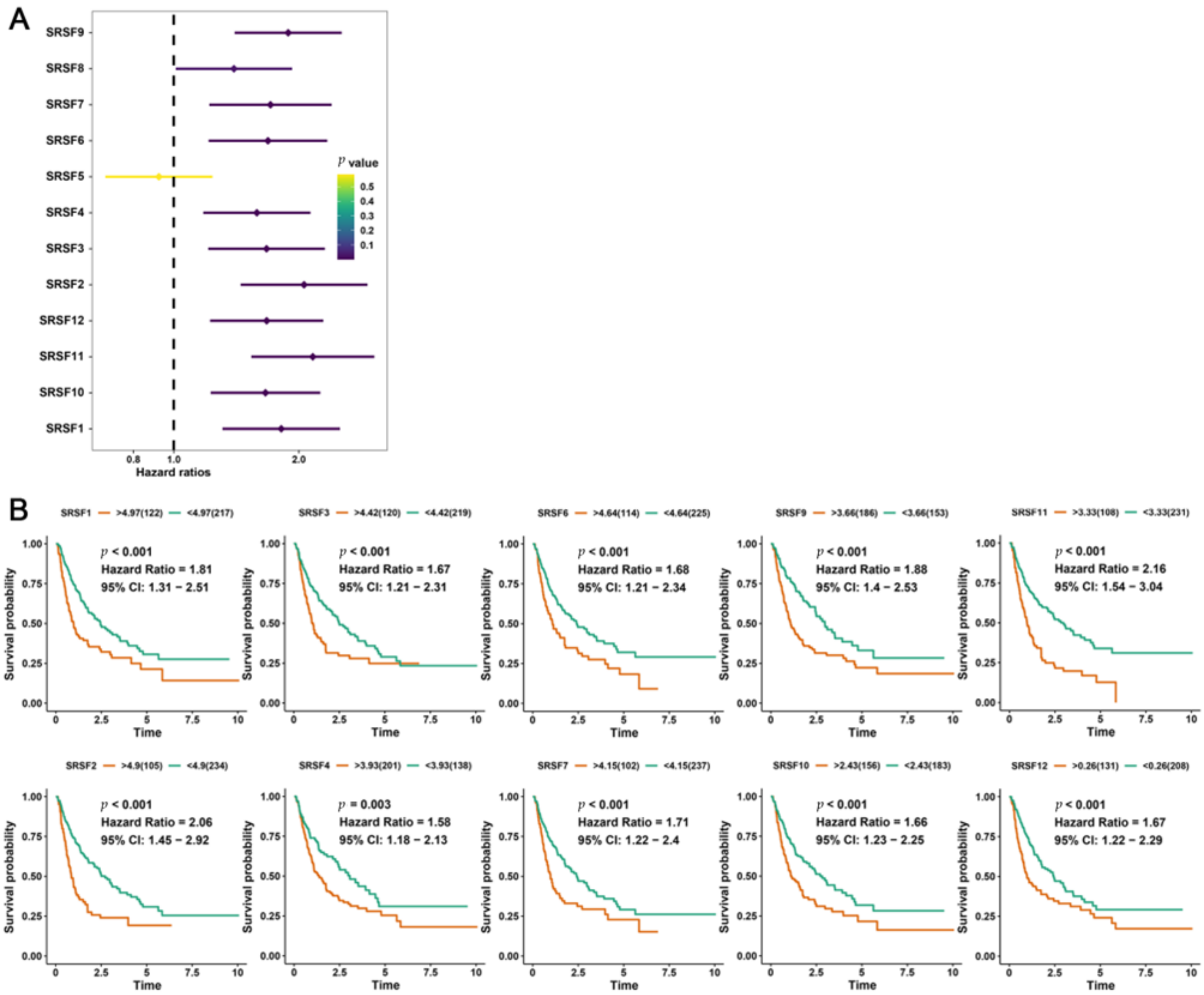

Figure S1. The prognostic analysis of SRSF family genes in HCC. (**A**) The progression-free survival (PFS) analysis of SRSF family genes in HCC. (**B**) Kaplan-Meier curves of PFS in the aggregated HCC cohort are stratified by the optimal cut-off value of the indicated SRSF genes.

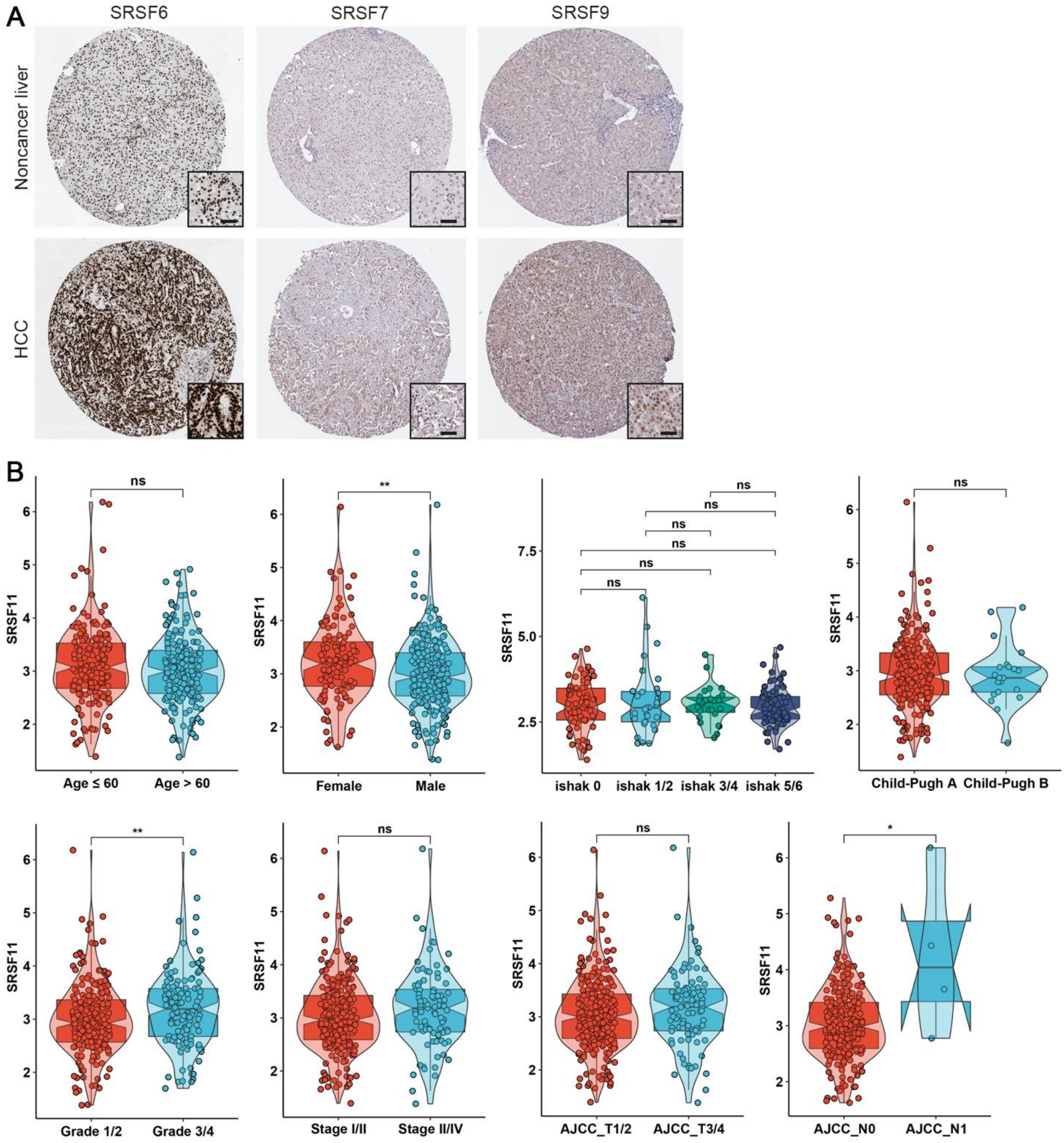

Figure S2. (**A**) Representative IHC images of SRSF family members in HCC and nontumor liver tissue from the Human Protein Atlas. Scale bar denotes 50 µm. (**B**) Correlation analysis of SRSF11 expression and clinicopathological parameters of HCC patients in TCGA-LIHC cohort. Student's *t* test was used for statistics. The asterisks represent the statistical p value (* $p < 0.05$ and ** $p < 0.01$). ns, no significance.

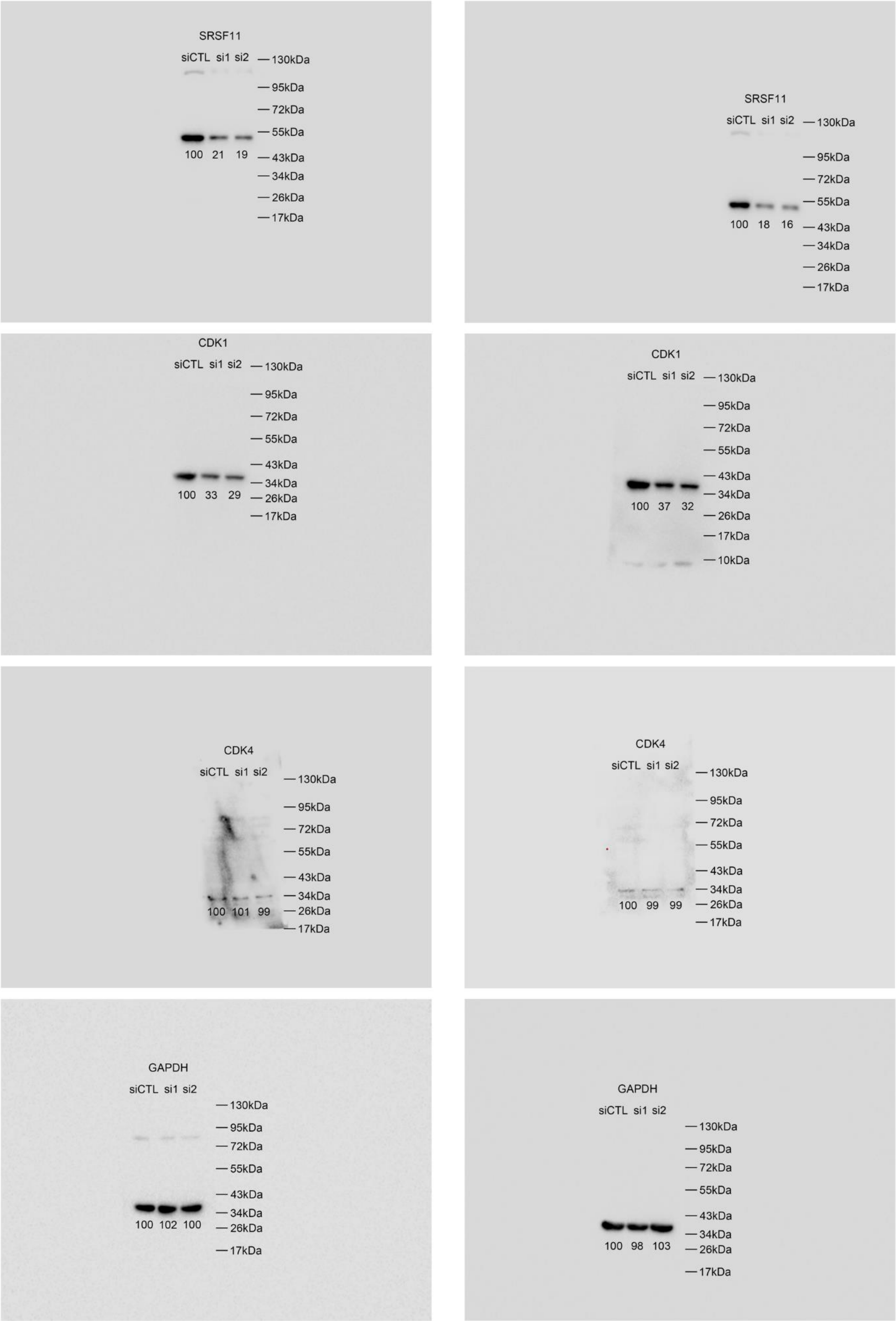

Figure S3. Uncropped Western blot images.

2. Supplementary Tables

Table S1. Correlations of SRSF11 expression with clinicopathological parameters.

Clinical Features		SRSF11 Expression		p Value
		High	Low	
Gender	Male	10	11	1.000
	Female	5	4	
Age (years)	>55	13	11	0.651
	≤55	2	4	
HBV DNA	Positive	14	10	0.169
	Negative	1	5	
History of alcoholism	With	4	3	1.000
	Without	11	12	
Cirrhosis	With	14	12	0.598
	Without	1	3	
BCLC stage	A+B	12	14	0.598
	C+D	3	1	
AJCC stage	I-II	13	14	1.000
	III	2	1	
Tumor size (cm)	>5	10	2	**0.009**
	≤5	6	13	
Tumor number	1	8	14	**0.035**
	>1	7	1	
Differentiation	G1-G2	10	12	0.682
	G3	5	3	
MVI	Present	5	2	0.340
	Absent	10	13	
Satellite lesions	Present	6	2	0.215
	Absent	9	13	
AFP	Normal	3	3	1.000
	Abnormal	12	12	

BCLC, barcelona clinic liver cancer; AJCC, american joint committee on cancer; MVI, microvascular invasion; AFP, alpha fetoprotein.

Table S2. Primary antibodies used in this study.

Antigens	Manufacturer	Catalog Number	Application
SRSF11	Abclonal	A18404	1:800 for WB, 1:100 for IHC
CDK1	Proteintech	19532-1-AP	1:800 for WB
CDK4	Proteintech	11026-1-AP	1:800 for WB
GAPDH	Proteintech	60004-1-Ig	1:2500 for WB

WB, western blot; IHC, immunohistochemistry.

Table S3. Sequences of siRNAs in this study.

Primer Names	Sequences
siSRSF11-1 sense	5′-GGUUGUUCACUUAUUGUUAUU-3′
siSRSF11-1 anti-sense	5′-UAACAAUAAGUGAACAACCUA-3′
siSRSF11-2 sense	5′-GGAGAAGAAGAAAGAUAAAGA-3′
siSRSF11-2 anti-sense	5′-UUUAUCUUUCUUCUUCUCCUU-3′

Table S4. The qRT-PCR primers used in this study.

Primer Names	Species	Sequences
β-actin forward	Human	5′-GAAGATCAAGATCATTGCTCC-3′
β-actin reverse	Human	5′-TACTCCTGCTTGCTGATCCA-3′
SRSF11 forward	Human	5′-ATGAGCAACACTACCGTCGTC-3′
SRSF11 reverse	Human	5′-GGGAGACATTAGTCACCTGGAT-3′

Table S5. List of SRSF family related genes with correlation >0.6.

SRSF Family	Gene	Correlation	*p* Value
SRSF11	PRPF38B	0.834080969	5.07E-161
SRSF9	CDK4	0.827333838	3.46E-156
SRSF9	RAN	0.808522959	9.49E-144
SRSF11	FNBP4	0.794371272	2.87E-135
SRSF9	TUBA1B	0.787801427	1.49E-131
SRSF11	NKTR	0.778973402	9.10E-127
SRSF11	MYSM1	0.77882016	1.10E-126
SRSF9	RAB35	0.777321368	6.77E-126
SRSF11	CCDC14	0.775153483	9.17E-125
SRSF11	ZRANB2	0.775018622	1.08E-124
SRSF3	TRA2B	0.773538779	6.27E-124
SRSF11	PRPF39	0.771334901	8.43E-123
SRSF11	TIA1	0.769266679	9.41E-122
SRSF6	RBM39	0.767680285	5.89E-121
SRSF9	DENR	0.767482077	7.39E-121
SRSF11	CCNT2	0.767396304	8.16E-121
SRSF11	MTF2	0.766379627	2.62E-120
SRSF1	LUC7L3	0.763044572	1.15E-118
SRSF10	USP48	0.760720698	1.54E-117
SRSF9	RNF34	0.760364218	2.29E-117
SRSF10	HP1BP3	0.758856577	1.22E-116
SRSF2	CEP95	0.757936652	3.34E-116
SRSF11	DMTF1	0.753078996	6.46E-114
SRSF1	HNRNPA2B1	0.752926118	7.61E-114
SRSF11	SREK1	0.752540079	1.15E-113
SRSF9	DIABLO	0.750854599	6.92E-113
SRSF10	PRPF38B	0.7474775	2.41E-111
SRSF11	ZDHHC17	0.746708169	5.37E-111
SRSF4	SRRM1	0.745657506	1.60E-110
SRSF11	CREBZF	0.74346536	1.52E-109
SRSF1	HNRNPA3	0.740143155	4.45E-108
SRSF9	NUP37	0.739529922	8.24E-108
SRSF9	UBE2N	0.737893983	4.24E-107
SRSF1	TCERG1	0.737480603	6.40E-107
SRSF2	PGS1	0.73650466	1.69E-106
SRSF11	ZNF326	0.735891689	3.10E-106
SRSF1	U2SURP	0.734882381	8.38E-106
SRSF11	PNISR	0.734639458	1.06E-105
SRSF2	LUC7L3	0.734161341	1.70E-105
SRSF11	RBM25	0.732892663	5.89E-105
SRSF1	TRA2B	0.732087212	1.29E-104

SRSF11	SF3B1	0.730993973	3.72E-104
SRSF9	MAPKAPK5	0.728679126	3.45E-103
SRSF2	HNRNPA3	0.728401944	4.49E-103
SRSF10	SRRM1	0.728380556	4.59E-103
SRSF11	NPHP3	0.728012345	6.52E-103
SRSF11	REV1	0.727110648	1.54E-102
SRSF3	RBMX	0.726972821	1.75E-102
SRSF1	CPSF6	0.726733616	2.20E-102
SRSF11	USP33	0.726239658	3.51E-102
SRSF11	LRIG2	0.725296621	8.56E-102
SRSF2	NUP85	0.724915469	1.23E-101
SRSF1	CNOT6	0.723927289	3.10E-101
SRSF3	KHDRBS1	0.723510168	4.58E-101
SRSF1	NAA40	0.723405358	5.05E-101
SRSF1	SFPQ	0.723037366	7.12E-101
SRSF1	RFWD3	0.722821857	8.71E-101
SRSF10	FOXJ3	0.722639849	1.03E-100
SRSF11	HNRNPH1	0.7224533	1.23E-100
SRSF10	SFPQ	0.720890813	5.23E-100
SRSF11	RBM39	0.720657609	6.49E-100
SRSF10	RBBP4	0.720339076	8.70E-100
SRSF3	RIOK1	0.719376493	2.11E-99
SRSF1	ATAD5	0.718920508	3.21E-99
SRSF9	SSRP1	0.718847866	3.43E-99
SRSF10	PNRC2	0.718164816	6.40E-99
SRSF11	TMX3	0.717797884	8.95E-99
SRSF2	GGA3	0.717508975	1.16E-98
SRSF1	CBX1	0.716397634	3.20E-98
SRSF1	CCAR1	0.715813599	5.43E-98
SRSF1	CCDC43	0.715262652	8.92E-98
SRSF3	HNRNPA1	0.714578089	1.65E-97
SRSF1	KPNB1	0.713656727	3.78E-97
SRSF1	DDX42	0.713155257	5.92E-97
SRSF1	TOPBP1	0.713091429	6.27E-97
SRSF1	ZNF207	0.712840538	7.84E-97
SRSF1	MTF2	0.712689965	8.97E-97
SRSF3	PTMA	0.712585101	9.85E-97
SRSF1	HNRNPL	0.712472022	1.09E-96
SRSF3	HNRNPL	0.712264321	1.31E-96
SRSF3	PRIM2	0.711327024	3.02E-96
SRSF2	POLG2	0.710822007	4.72E-96
SRSF9	CBX1	0.710717809	5.17E-96
SRSF11	FOXJ3	0.710374927	7.00E-96
SRSF11	RBBP6	0.71023152	7.95E-96
SRSF10	MTF2	0.709049563	2.25E-95
SRSF9	CCT2	0.708954795	2.45E-95
SRSF1	ILF3	0.708385339	4.03E-95
SRSF1	CENPK	0.707327544	1.02E-94
SRSF1	BUB1B	0.707311749	1.03E-94
SRSF10	OTUD3	0.706018706	3.17E-94
SRSF1	HNRNPU	0.705708048	4.15E-94

SRSF2	DBF4B	0.705558847	4.72E-94
SRSF1	CEP95	0.705441491	5.23E-94
SRSF2	TCERG1	0.705401401	5.41E-94
SRSF3	HNRNPD	0.705363386	5.59E-94
SRSF6	PRPF39	0.705080907	7.14E-94
SRSF1	WDHD1	0.704880765	8.48E-94
SRSF11	ANKRD13C	0.704702374	9.90E-94
SRSF2	DDX42	0.704120061	1.63E-93
SRSF11	MIER1	0.703853249	2.06E-93
SRSF9	ANAPC5	0.703679759	2.39E-93
SRSF2	U2SURP	0.703390302	3.06E-93
SRSF3	MCM3	0.702614589	5.94E-93
SRSF9	PRKAG1	0.702531968	6.38E-93
SRSF2	HNRNPA2B1	0.702508874	6.50E-93
SRSF1	NCAPD2	0.702237397	8.20E-93
SRSF1	ACTL6A	0.702195636	8.50E-93
SRSF9	TUBA1C	0.701202782	1.98E-92
SRSF2	NAA40	0.701095383	2.17E-92
SRSF2	SUPT7L	0.700976223	2.40E-92
SRSF10	ZMYM1	0.700840198	2.69E-92
SRSF9	SAE1	0.70072842	2.96E-92
SRSF3	HNRNPC	0.70044657	3.76E-92
SRSF1	U2AF2	0.699740095	6.83E-92
SRSF9	SART3	0.699605086	7.66E-92
SRSF1	DBF4B	0.699572648	7.87E-92
SRSF11	TCERG1	0.699434075	8.85E-92
SRSF10	ZMYM4	0.698734354	1.60E-91
SRSF11	CCNL1	0.698135632	2.64E-91
SRSF2	SFPQ	0.698113769	2.69E-91
SRSF11	PKN2	0.69807444	2.78E-91
SRSF11	PTBP2	0.697656852	3.94E-91
SRSF2	TRA2B	0.696882264	7.53E-91
SRSF11	ZNF644	0.696165373	1.37E-90
SRSF3	HNRNPA3	0.696052093	1.50E-90
SRSF1	KIF18B	0.695966534	1.61E-90
SRSF11	RBM33	0.695651696	2.10E-90
SRSF1	FANCD2	0.694909582	3.88E-90
SRSF4	USP48	0.694733267	4.49E-90
SRSF2	KNTC1	0.694439433	5.73E-90
SRSF11	NR2C2	0.694304276	6.40E-90
SRSF1	KIF23	0.694153698	7.25E-90
SRSF2	PRPF39	0.6941333	7.37E-90
SRSF11	SETD5	0.6935606	1.18E-89
SRSF10	SF3B1	0.693403673	1.35E-89
SRSF1	KNTC1	0.693326753	1.43E-89
SRSF2	ATAD5	0.693111472	1.71E-89
SRSF9	SMARCB1	0.692917862	2.01E-89
SRSF9	PPHLN1	0.692594879	2.61E-89
SRSF2	IQCB1	0.692510103	2.80E-89
SRSF1	PGS1	0.692249293	3.47E-89
SRSF11	U2SURP	0.691894387	4.64E-89

SRSF11	STX16	0.69150473	6.38E-89
SRSF1	YEATS2	0.691361075	7.17E-89
SRSF10	EIF4G3	0.691128178	8.67E-89
SRSF1	MITD1	0.691087854	8.96E-89
SRSF10	PPP1R12A	0.691008949	9.56E-89
SRSF7	TRA2B	0.690376888	1.60E-88
SRSF1	CENPF	0.690276619	1.73E-88
SRSF1	NUF2	0.689786651	2.58E-88
SRSF1	UBA2	0.689094884	4.52E-88
SRSF1	KIF18A	0.689088064	4.54E-88
SRSF1	ECT2	0.688899058	5.29E-88
SRSF1	HNRNPH3	0.68884734	5.52E-88
SRSF10	RBM12B	0.688839314	5.55E-88
SRSF11	MON2	0.688652999	6.46E-88
SRSF10	CAPZA1	0.688493121	7.34E-88
SRSF2	ILF3	0.688270955	8.79E-88
SRSF1	ARL6IP6	0.68811687	9.95E-88
SRSF11	RLF	0.688076934	1.03E-87
SRSF1	RCC2	0.687985335	1.11E-87
SRSF1	LMNB1	0.687947183	1.14E-87
SRSF1	KIF11	0.687752516	1.33E-87
SRSF1	NCAPH	0.687734961	1.35E-87
SRSF1	NUP107	0.687638286	1.46E-87
SRSF11	INO80D	0.687626037	1.48E-87
SRSF3	HNRNPM	0.687067362	2.31E-87
SRSF1	TTF2	0.68706627	2.31E-87
SRSF9	SET	0.686994964	2.45E-87
SRSF9	HNRNPM	0.686803838	2.86E-87
SRSF11	PAN3	0.686302791	4.26E-87
SRSF9	DNAJC9	0.685853977	6.10E-87
SRSF2	PRPF38B	0.685831052	6.21E-87
SRSF2	HNRNPH3	0.685736816	6.70E-87
SRSF1	GTF3C2	0.685671861	7.05E-87
SRSF1	CDK2	0.685430478	8.55E-87
SRSF10	RLF	0.685307796	9.43E-87
SRSF3	MAPRE1	0.685231398	1.00E-86
SRSF2	CSNK1D	0.68514256	1.08E-86
SRSF10	FUBP1	0.684785779	1.43E-86
SRSF1	SUPT7L	0.684457103	1.85E-86
SRSF1	E2F6	0.684450313	1.86E-86
SRSF10	RBBP6	0.684320929	2.07E-86
SRSF1	PRPF39	0.684186214	2.30E-86
SRSF11	RSRC2	0.684014326	2.63E-86
SRSF2	ZNF207	0.683996522	2.67E-86
SRSF1	HJURP	0.683972849	2.72E-86
SRSF1	CEP55	0.683838903	3.03E-86
SRSF2	MTF2	0.683778063	3.17E-86
SRSF9	DDX23	0.683759875	3.22E-86
SRSF2	RBM39	0.683722344	3.32E-86
SRSF1	NCBP2	0.683602837	3.65E-86
SRSF11	NR2C1	0.683326228	4.54E-86

SRSF1	CASP2	0.683304548	4.62E-86
SRSF1	PRPF38A	0.683203159	5.00E-86
SRSF1	HELLS	0.683011962	5.82E-86
SRSF1	TDG	0.682668936	7.62E-86
SRSF1	ORC6	0.682539463	8.44E-86
SRSF1	RNF34	0.682355091	9.76E-86
SRSF10	HNRNPR	0.682290718	1.03E-85
SRSF4	HP1BP3	0.682096886	1.20E-85
SRSF10	ZDHHC17	0.682014584	1.28E-85
SRSF1	PRPF38B	0.681826359	1.48E-85
SRSF1	COIL	0.681654913	1.69E-85
SRSF6	FNBP4	0.681630783	1.72E-85
SRSF11	RSBN1	0.681096656	2.62E-85
SRSF2	ARL6IP6	0.680966435	2.90E-85
SRSF2	E2F6	0.680556944	3.99E-85
SRSF2	HNRNPH1	0.680168537	5.41E-85
SRSF10	EYA3	0.679751289	7.49E-85
SRSF2	NUP107	0.679749561	7.50E-85
SRSF1	UHRF1	0.679516583	8.99E-85
SRSF9	YWHAQ	0.679487485	9.19E-85
SRSF10	SMC4	0.679308923	1.06E-84
SRSF1	RAD51AP1	0.679303813	1.06E-84
SRSF11	ZNF638	0.679273203	1.09E-84
SRSF10	CRLF3	0.678961977	1.38E-84
SRSF6	RBM25	0.678936231	1.41E-84
SRSF1	POLQ	0.678716373	1.67E-84
SRSF2	HNRNPD	0.67835839	2.21E-84
SRSF1	XRCC2	0.678204518	2.49E-84
SRSF1	PLK4	0.678200667	2.50E-84
SRSF2	MYO19	0.678163461	2.57E-84
SRSF1	IQCB1	0.678121343	2.65E-84
SRSF1	CDC7	0.677867989	3.23E-84
SRSF11	ZMYM1	0.677855179	3.26E-84
SRSF3	FUS	0.677569559	4.06E-84
SRSF9	CCT5	0.677379128	4.71E-84
SRSF1	SPATS2	0.677357928	4.78E-84
SRSF1	BRPF1	0.677127508	5.71E-84
SRSF1	DNMT1	0.677091587	5.87E-84
SRSF10	SPEN	0.677075284	5.95E-84
SRSF11	ZCCHC8	0.676864241	7.00E-84
SRSF1	FANCL	0.676253736	1.12E-83
SRSF3	HNRNPU	0.676219143	1.15E-83
SRSF11	ZNF354B	0.676068468	1.29E-83
SRSF2	KIF18B	0.675876641	1.49E-83
SRSF3	HNRNPA0	0.675687546	1.73E-83
SRSF2	DDX55	0.675495064	2.00E-83
SRSF1	ARHGAP11A	0.675375506	2.19E-83
SRSF9	GTPBP4	0.675168719	2.57E-83
SRSF3	HNRNPH3	0.674920737	3.11E-83
SRSF2	BRPF1	0.674840985	3.30E-83
SRSF1	SMARCD1	0.674760523	3.51E-83

SRSF10	VRK2	0.674585586	4.01E-83
SRSF6	TIA1	0.674538242	4.16E-83
SRSF10	SETD5	0.674475187	4.36E-83
SRSF1	RBMX	0.674351805	4.79E-83
SRSF2	HELLS	0.674208257	5.35E-83
SRSF1	WDR75	0.674091529	5.84E-83
SRSF1	C4orf46	0.674008399	6.23E-83
SRSF2	GTF3C2	0.673879093	6.87E-83
SRSF10	S100PBP	0.673751411	7.57E-83
SRSF2	HNRNPL	0.673475403	9.34E-83
SRSF10	TRA2B	0.673370136	1.01E-82
SRSF10	USP33	0.673370027	1.01E-82
SRSF11	ZNF700	0.673063137	1.28E-82
SRSF10	ZRANB2	0.672863465	1.49E-82
SRSF1	HNRNPD	0.672856724	1.49E-82
SRSF1	DCAF16	0.672387299	2.13E-82
SRSF3	THUMPD2	0.672383968	2.14E-82
SRSF1	MTBP	0.672344051	2.20E-82
SRSF10	RCC2	0.672215861	2.42E-82
SRSF9	METAP2	0.67218128	2.49E-82
SRSF10	CCAR1	0.67209067	2.67E-82
SRSF1	RAD51D	0.671741468	3.47E-82
SRSF2	CENPK	0.671685706	3.62E-82
SRSF9	AACS	0.671598499	3.86E-82
SRSF1	KIF20A	0.671512105	4.12E-82
SRSF1	FANCB	0.671454871	4.31E-82
SRSF11	LUC7L3	0.67124713	5.03E-82
SRSF7	WDR75	0.671214772	5.16E-82
SRSF1	MMS22L	0.671133663	5.48E-82
SRSF3	SFPQ	0.670945631	6.32E-82
SRSF2	WDR75	0.670865388	6.71E-82
SRSF1	RBM12B	0.670828217	6.90E-82
SRSF2	U2AF2	0.670666188	7.79E-82
SRSF9	PPP1CC	0.670531586	8.62E-82
SRSF1	CRLF3	0.670504564	8.80E-82
SRSF9	GMPS	0.670190107	1.11E-81
SRSF10	MYSM1	0.670187208	1.12E-81
SRSF4	RLF	0.670050692	1.24E-81
SRSF1	ERCC3	0.670037523	1.25E-81
SRSF1	STIL	0.669733951	1.57E-81
SRSF1	GTSE1	0.669732793	1.57E-81
SRSF2	EWSR1	0.669625449	1.70E-81
SRSF9	CORO1C	0.669595637	1.74E-81
SRSF9	CDK2AP1	0.669560109	1.79E-81
SRSF3	NXT1	0.669337299	2.11E-81
SRSF11	USP24	0.669333164	2.12E-81
SRSF10	NR2C2	0.669333128	2.12E-81
SRSF1	TLK2	0.669019262	2.67E-81
SRSF3	CDCA4	0.668778667	3.20E-81
SRSF1	TARDBP	0.668411368	4.21E-81
SRSF3	CBX3	0.668167651	5.04E-81

SRSF10	CCDC14	0.668061898	5.46E-81
SRSF1	GINS1	0.667920542	6.06E-81
SRSF11	HEATR5B	0.667905007	6.13E-81
SRSF10	NAA40	0.667439482	8.66E-81
SRSF1	GGA3	0.667257673	9.91E-81
SRSF10	ARL13B	0.667111523	1.10E-80
SRSF2	ORC6	0.666931983	1.26E-80
SRSF2	MITD1	0.666922419	1.27E-80
SRSF1	RBL1	0.666790847	1.40E-80
SRSF1	MSH2	0.666532907	1.70E-80
SRSF2	TLK2	0.666433716	1.82E-80
SRSF11	RBM5	0.666407527	1.86E-80
SRSF1	HEATR6	0.666338727	1.96E-80
SRSF9	DHX37	0.666220098	2.14E-80
SRSF6	STX16	0.665978722	2.55E-80
SRSF10	ATM	0.665775278	2.97E-80
SRSF2	NUF2	0.6655393	3.53E-80
SRSF2	CLK2	0.665497052	3.64E-80
SRSF10	DYNC1LI2	0.665372672	3.99E-80
SRSF1	KIAA1841	0.665265706	4.31E-80
SRSF2	S100PBP	0.665196059	4.54E-80
SRSF2	HMGB2	0.66509636	4.89E-80
SRSF1	RPS6KB1	0.66498175	5.32E-80
SRSF10	G2E3	0.664794466	6.10E-80
SRSF3	ACTL6A	0.664682421	6.62E-80
SRSF2	RPS6KB1	0.664614719	6.96E-80
SRSF2	CCDC43	0.664583144	7.12E-80
SRSF2	RCC2	0.66438128	8.26E-80
SRSF3	ARL6IP6	0.664210407	9.36E-80
SRSF1	CDCA4	0.664043237	1.06E-79
SRSF1	SASS6	0.66402135	1.08E-79
SRSF9	KHDRBS1	0.663984516	1.10E-79
SRSF1	MELK	0.663724536	1.34E-79
SRSF2	TARDBP	0.66364114	1.42E-79
SRSF10	TTF2	0.663609369	1.45E-79
SRSF11	ZMYM4	0.663592824	1.47E-79
SRSF10	PAPOLG	0.66358905	1.47E-79
SRSF2	BUB1B	0.663385792	1.71E-79
SRSF9	PLK1	0.663212479	1.94E-79
SRSF1	MYO19	0.66316875	2.00E-79
SRSF11	SRRM1	0.663151667	2.03E-79
SRSF9	DDX54	0.663130123	2.06E-79
SRSF2	THOC1	0.662576491	3.09E-79
SRSF9	PWP1	0.662543268	3.16E-79
SRSF10	SMCHD1	0.662529684	3.19E-79
SRSF1	IFT80	0.662471059	3.33E-79
SRSF2	FANCD2	0.662442717	3.40E-79
SRSF2	CDK2	0.66240302	3.50E-79
SRSF10	PPP1R8	0.662334762	3.68E-79
SRSF1	KHDRBS1	0.662276674	3.84E-79
SRSF10	CASP8AP2	0.662234968	3.96E-79

SRSF1	ANLN	0.662221883	3.99E-79
SRSF1	H2AFY	0.662213799	4.02E-79
SRSF2	POLQ	0.662116555	4.31E-79
SRSF3	CCDC59	0.662011045	4.66E-79
SRSF9	ACTL6A	0.661861207	5.19E-79
SRSF7	HNRNPH3	0.661734303	5.69E-79
SRSF10	DR1	0.661710988	5.79E-79
SRSF2	LMNB1	0.661626607	6.15E-79
SRSF2	UNK	0.661602345	6.26E-79
SRSF10	ZBTB40	0.66150382	6.73E-79
SRSF9	TRAFD1	0.661186267	8.47E-79
SRSF10	NKTR	0.661033683	9.46E-79
SRSF1	MAML1	0.661012939	9.60E-79
SRSF2	OTUD3	0.660943356	1.01E-78
SRSF1	ZFC3H1	0.660806302	1.11E-78
SRSF1	NUP43	0.660778465	1.14E-78
SRSF9	NCL	0.660655953	1.24E-78
SRSF3	CHTOP	0.660448913	1.44E-78
SRSF1	STX6	0.660384689	1.51E-78
SRSF2	HEATR6	0.660365351	1.53E-78
SRSF9	SMARCD1	0.660294643	1.61E-78
SRSF1	MCM8	0.66008662	1.87E-78
SRSF10	PUM1	0.660012933	1.98E-78
SRSF2	HJURP	0.659939578	2.08E-78
SRSF9	COMMD2	0.659840288	2.24E-78
SRSF11	HP1BP3	0.659736509	2.41E-78
SRSF3	HDAC2	0.659629898	2.60E-78
SRSF11	NAA25	0.659605215	2.65E-78
SRSF3	TBC1D22B	0.659537304	2.78E-78
SRSF1	TOP2A	0.659456186	2.95E-78
SRSF1	S100PBP	0.659294522	3.31E-78
SRSF1	DNM1L	0.659285609	3.34E-78
SRSF1	SMC4	0.659274256	3.36E-78
SRSF4	S100PBP	0.659175616	3.61E-78
SRSF3	NOP56	0.659145982	3.69E-78
SRSF10	SP1	0.658834822	4.61E-78
SRSF10	ZC3H7A	0.658725496	4.99E-78
SRSF9	HNRNPC	0.658695382	5.10E-78
SRSF11	STXBP3	0.658684216	5.14E-78
SRSF1	DVL3	0.658589448	5.50E-78
SRSF1	METTL4	0.658513784	5.80E-78
SRSF1	SART3	0.658439915	6.12E-78
SRSF11	ZNF518A	0.658432608	6.15E-78
SRSF9	MRPL42	0.658414984	6.23E-78
SRSF2	ACIN1	0.65835051	6.52E-78
SRSF9	U2AF2	0.658237961	7.07E-78
SRSF9	NCAPH	0.658154892	7.50E-78
SRSF9	CPSF6	0.658101164	7.80E-78
SRSF2	SART3	0.658051111	8.08E-78
SRSF1	TRIP13	0.65780882	9.61E-78
SRSF1	MCM3	0.657760656	9.95E-78

SRSF9	PPP6R1	0.657733285	1.01E-77
SRSF2	ERCC3	0.657569473	1.14E-77
SRSF10	HIPK1	0.65751981	1.18E-77
SRSF1	DEPDC1B	0.657406212	1.28E-77
SRSF11	CHD2	0.657329091	1.35E-77
SRSF3	ILF2	0.657320356	1.36E-77
SRSF1	POLA1	0.657290931	1.39E-77
SRSF10	PKN2	0.657177937	1.51E-77
SRSF1	RBBP4	0.657173511	1.51E-77
SRSF6	RBM5	0.656886974	1.86E-77
SRSF2	PRPF38A	0.656868529	1.88E-77
SRSF9	GATC	0.656821265	1.94E-77
SRSF10	PNISR	0.656803561	1.97E-77
SRSF2	CDCA4	0.65655269	2.35E-77
SRSF1	TRIM59	0.656403289	2.62E-77
SRSF1	ARMC8	0.656329092	2.76E-77
SRSF2	RAD54L	0.656166292	3.10E-77
SRSF10	U2SURP	0.655954552	3.60E-77
SRSF1	ZNF248	0.655898381	3.75E-77
SRSF1	GPD2	0.65583683	3.91E-77
SRSF11	S100PBP	0.655722506	4.24E-77
SRSF9	KPNA2	0.655680807	4.37E-77
SRSF2	TDG	0.655622858	4.55E-77
SRSF2	CDC7	0.65549991	4.97E-77
SRSF11	MED23	0.655453363	5.14E-77
SRSF11	IFT80	0.655429428	5.22E-77
SRSF9	GRB2	0.655414931	5.28E-77
SRSF2	ANKLE2	0.655294721	5.75E-77
SRSF10	CCNT2	0.655113732	6.53E-77
SRSF9	DCTN2	0.655079184	6.69E-77
SRSF11	GPATCH8	0.654975871	7.20E-77
SRSF1	LSM14A	0.654853264	7.85E-77
SRSF10	ADAM17	0.654830762	7.98E-77
SRSF10	FNBP4	0.654808486	8.10E-77
SRSF2	NCAPH	0.65464413	9.10E-77
SRSF1	KIF2C	0.654641492	9.12E-77
SRSF2	RBM5	0.654562705	9.64E-77
SRSF1	NCOA5	0.654495531	1.01E-76
SRSF1	ERCC6L	0.654457568	1.04E-76
SRSF1	BUB1	0.654348552	1.12E-76
SRSF10	CEP170	0.654309468	1.15E-76
SRSF10	STK4	0.654262216	1.19E-76
SRSF10	YTHDC1	0.654219599	1.23E-76
SRSF3	DEK	0.654155731	1.28E-76
SRSF1	NUSAP1	0.654138661	1.30E-76
SRSF1	PRC1	0.653744339	1.72E-76
SRSF4	YTHDF2	0.653673782	1.80E-76
SRSF2	FBXO5	0.653658764	1.82E-76
SRSF2	TIA1	0.653633556	1.86E-76
SRSF2	FANCL	0.653619451	1.87E-76
SRSF2	STX16	0.653453677	2.11E-76

SRSF2	FNBP4	0.65312285	2.66E-76
SRSF10	PPIL4	0.652783783	3.37E-76
SRSF3	FANCE	0.652769077	3.40E-76
SRSF2	EZH2	0.652751586	3.45E-76
SRSF10	BCLAF1	0.652746406	3.46E-76
SRSF9	PDCD10	0.652706408	3.56E-76
SRSF10	TOP1	0.652650845	3.70E-76
SRSF1	DBR1	0.652491109	4.14E-76
SRSF1	ZBED5	0.652480717	4.17E-76
SRSF9	TPM3	0.652443943	4.28E-76
SRSF11	THRAP3	0.652412264	4.37E-76
SRSF9	PRDM4	0.652254476	4.88E-76
SRSF2	KIF23	0.652194612	5.09E-76
SRSF11	ARGLU1	0.65216837	5.18E-76
SRSF1	ANKLE2	0.652164115	5.20E-76
SRSF1	ZWILCH	0.652047839	5.64E-76
SRSF1	RBM39	0.651944021	6.06E-76
SRSF7	MITD1	0.651925958	6.14E-76
SRSF10	SP3	0.651810469	6.66E-76
SRSF1	PHIP	0.651740821	6.99E-76
SRSF1	DDX55	0.651683842	7.27E-76
SRSF1	BRCA1	0.651626133	7.57E-76
SRSF1	VRK2	0.651596803	7.73E-76
SRSF10	NEDD1	0.651522378	8.14E-76
SRSF3	EEF1E1	0.651481181	8.38E-76
SRSF1	ZBTB34	0.651406315	8.83E-76
SRSF9	MORF4L1	0.651342251	9.23E-76
SRSF2	DNMT1	0.651333647	9.29E-76
SRSF10	PHACTR4	0.651319072	9.38E-76
SRSF1	ZNF142	0.651289723	9.58E-76
SRSF1	MCM6	0.651277085	9.66E-76
SRSF2	UTP6	0.651156037	1.05E-75
SRSF3	KIFC1	0.651112729	1.08E-75
SRSF11	PHF12	0.651105943	1.09E-75
SRSF2	KPNB1	0.651056092	1.13E-75
SRSF1	TIA1	0.651030632	1.15E-75
SRSF1	POLD3	0.65076851	1.38E-75
SRSF1	CENPE	0.650628719	1.52E-75
SRSF1	NCAPG	0.650608915	1.54E-75
SRSF3	ZNF207	0.650438972	1.73E-75
SRSF6	ASXL1	0.650309404	1.89E-75
SRSF2	PHF12	0.650307262	1.90E-75
SRSF10	HNRNPA2B1	0.65026409	1.95E-75
SRSF1	PRKCI	0.650210232	2.03E-75
SRSF10	RFWD3	0.650188865	2.06E-75
SRSF1	CDK1	0.650178807	2.07E-75
SRSF3	DUSP12	0.650110468	2.17E-75
SRSF1	HDAC2	0.650103192	2.19E-75
SRSF2	PLK4	0.64993604	2.45E-75
SRSF2	PMS1	0.6499082	2.50E-75
SRSF1	KIF15	0.649714256	2.86E-75

SRSF2	ACTL6A	0.649631261	3.03E-75
SRSF9	MAPRE1	0.649451243	3.43E-75
SRSF3	EWSR1	0.649432991	3.48E-75
SRSF10	IQCB1	0.649366481	3.64E-75
SRSF2	TOPBP1	0.649309708	3.79E-75
SRSF1	FBXO5	0.649282512	3.86E-75
SRSF1	TTK	0.649277269	3.87E-75
SRSF3	SNRPD1	0.649264093	3.91E-75
SRSF9	ECT2	0.649125247	4.30E-75
SRSF11	PNN	0.649005953	4.67E-75
SRSF1	CCDC14	0.648251225	7.87E-75
SRSF1	FANCG	0.648138729	8.50E-75
SRSF1	POLG2	0.648113883	8.65E-75
SRSF9	CSNK2A1	0.64804671	9.06E-75
SRSF2	SF3B1	0.647865738	1.03E-74
SRSF1	RFC4	0.647821419	1.06E-74
SRSF10	IFT80	0.64774195	1.12E-74
SRSF1	XPO1	0.647731493	1.13E-74
SRSF4	EYA3	0.64753485	1.29E-74
SRSF9	ZNF664	0.647457918	1.36E-74
SRSF1	SENP1	0.647416187	1.40E-74
SRSF7	RBM39	0.64741231	1.40E-74
SRSF10	IKZF4	0.647117704	1.72E-74
SRSF1	RYK	0.647098178	1.74E-74
SRSF9	NUP107	0.647084567	1.75E-74
SRSF9	ARPC2	0.646972215	1.90E-74
SRSF11	LRRC40	0.646827313	2.09E-74
SRSF1	ADNP	0.646799662	2.13E-74
SRSF2	FANCG	0.64676081	2.19E-74
SRSF10	THRAP3	0.646665868	2.34E-74
SRSF11	YTHDC1	0.646615504	2.42E-74
SRSF3	LMNB2	0.646489321	2.64E-74
SRSF1	COMMD2	0.646424864	2.76E-74
SRSF1	THOC1	0.646392207	2.82E-74
SRSF2	FOXK2	0.646345579	2.91E-74
SRSF7	PMS1	0.646320104	2.96E-74
SRSF1	CDC6	0.646293988	3.02E-74
SRSF3	BRIX1	0.64625794	3.09E-74
SRSF6	DMTF1	0.646184073	3.25E-74
SRSF9	UTP20	0.646159142	3.31E-74
SRSF9	HNRNPR	0.646157353	3.31E-74
SRSF9	SNRNP40	0.646115355	3.41E-74
SRSF2	SMARCD1	0.646094625	3.46E-74
SRSF11	TTC14	0.64604693	3.57E-74
SRSF4	CDK11B	0.646040108	3.59E-74
SRSF10	SCLT1	0.646036941	3.60E-74
SRSF2	CBX1	0.645901747	3.94E-74
SRSF9	ANKLE2	0.645822485	4.16E-74
SRSF9	TRIP13	0.645798122	4.23E-74
SRSF3	UBA2	0.645787219	4.26E-74
SRSF10	TAF1B	0.645782266	4.28E-74

SRSF2	SRRT	0.645637202	4.72E-74
SRSF1	ABI2	0.645636237	4.73E-74
SRSF2	RBM17	0.645593145	4.87E-74
SRSF2	CRLF3	0.645532062	5.08E-74
SRSF1	LMNB2	0.645480702	5.26E-74
SRSF3	NOP58	0.645421097	5.48E-74
SRSF1	MKI67	0.645345531	5.76E-74
SRSF1	NEDD1	0.64528711	6.00E-74
SRSF1	ACIN1	0.645173351	6.48E-74
SRSF10	BAZ2A	0.645082799	6.89E-74
SRSF1	RAD54L	0.64491379	7.74E-74
SRSF11	ASXL1	0.644913534	7.74E-74
SRSF1	MCM10	0.644886982	7.88E-74
SRSF11	PMS1	0.644879297	7.92E-74
SRSF11	POGZ	0.644877728	7.93E-74
SRSF1	SKA3	0.644869747	7.97E-74
SRSF7	THUMPD2	0.644843027	8.12E-74
SRSF10	CPSF6	0.644833312	8.17E-74
SRSF3	RCC2	0.644715473	8.85E-74
SRSF1	POGK	0.644688111	9.02E-74
SRSF1	QSER1	0.644684258	9.04E-74
SRSF1	HAUS3	0.644662102	9.18E-74
SRSF2	WDHD1	0.644389513	1.10E-73
SRSF7	HNRNPD	0.644381879	1.11E-73
SRSF1	CENPO	0.644298121	1.18E-73
SRSF9	LMNB2	0.644240478	1.22E-73
SRSF10	TDG	0.644178441	1.28E-73
SRSF11	ZNF337	0.6439611	1.48E-73
SRSF9	MCM2	0.643851604	1.59E-73
SRSF10	DNMT1	0.643835491	1.61E-73
SRSF1	CKAP5	0.643718754	1.74E-73
SRSF11	RICTOR	0.643647009	1.83E-73
SRSF9	PPP5C	0.643574462	1.92E-73
SRSF1	ZWINT	0.643505777	2.01E-73
SRSF1	PTMA	0.643406217	2.15E-73
SRSF1	FANCI	0.643283115	2.34E-73
SRSF11	BRD1	0.643239462	2.41E-73
SRSF1	ORC2	0.643176912	2.51E-73
SRSF2	KIF11	0.643111201	2.63E-73
SRSF2	CCDC14	0.642991511	2.85E-73
SRSF2	ATXN7L3	0.642949285	2.93E-73
SRSF11	ZNF621	0.64286696	3.10E-73
SRSF10	HNRNPK	0.642858605	3.12E-73
SRSF1	NEK2	0.64285207	3.13E-73
SRSF2	SMPD4	0.642830497	3.18E-73
SRSF1	INCENP	0.642823362	3.19E-73
SRSF1	DHX9	0.642804458	3.23E-73
SRSF1	MCM2	0.642759084	3.33E-73
SRSF1	SETD5	0.642735201	3.39E-73
SRSF10	ETNK1	0.642526113	3.90E-73
SRSF1	MASTL	0.642519077	3.92E-73

SRSF11	NAA40	0.642518667	3.92E-73
SRSF1	CHTOP	0.642431583	4.16E-73
SRSF2	RFWD3	0.64240826	4.22E-73
SRSF10	CDC7	0.642366634	4.34E-73
SRSF1	PPHLN1	0.64235191	4.39E-73
SRSF1	FUBP1	0.642255902	4.68E-73
SRSF9	GINS1	0.641988079	5.60E-73
SRSF11	LENG8	0.641704426	6.78E-73
SRSF9	LLPH	0.641442718	8.09E-73
SRSF10	TCERG1	0.641359667	8.55E-73
SRSF9	NCAPD2	0.641352789	8.59E-73
SRSF9	BUB1B	0.641341911	8.65E-73
SRSF6	ZNF638	0.641328523	8.73E-73
SRSF10	MIER1	0.641305786	8.87E-73
SRSF9	FOXM1	0.641304188	8.88E-73
SRSF11	BAZ2A	0.641257173	9.16E-73
SRSF2	ORC2	0.641232107	9.32E-73
SRSF9	ARL1	0.641202704	9.50E-73
SRSF2	NCAPD2	0.641127983	9.99E-73
SRSF1	LARP4B	0.641084898	1.03E-72
SRSF1	BUB3	0.640907515	1.16E-72
SRSF1	ORC1	0.640902408	1.16E-72
SRSF9	HNRNPA0	0.640887565	1.17E-72
SRSF10	C18orf25	0.640776263	1.26E-72
SRSF1	CDCA8	0.64068809	1.34E-72
SRSF1	CNOT3	0.640657371	1.37E-72
SRSF10	SART3	0.640645457	1.38E-72
SRSF1	PAPOLG	0.640578062	1.44E-72
SRSF1	PRR11	0.640548732	1.47E-72
SRSF1	USP39	0.640539676	1.48E-72
SRSF3	NRM	0.640489183	1.53E-72
SRSF9	NIPA2	0.640487334	1.53E-72
SRSF1	CDC27	0.640246333	1.80E-72
SRSF1	RAD18	0.640238957	1.81E-72
SRSF2	PIF1	0.640107052	1.98E-72
SRSF1	HSF2	0.640005174	2.12E-72
SRSF10	YTHDF2	0.639966254	2.17E-72
SRSF1	EXO1	0.639900335	2.27E-72
SRSF9	CCT4	0.639786628	2.45E-72
SRSF1	PMS1	0.63978431	2.46E-72
SRSF6	BRD1	0.63977517	2.47E-72
SRSF3	FANCG	0.639676889	2.64E-72
SRSF10	PHF6	0.639663699	2.66E-72
SRSF1	PLK1	0.639581404	2.81E-72
SRSF1	FANCF	0.639513837	2.94E-72
SRSF1	CENPA	0.639410238	3.15E-72
SRSF9	PDIA6	0.639355658	3.27E-72
SRSF10	FBXO30	0.639267998	3.47E-72
SRSF11	TET3	0.63923901	3.53E-72
SRSF1	EZH2	0.639145956	3.76E-72
SRSF11	TRIM33	0.639126316	3.81E-72

SRSF11	OGT	0.639081977	3.92E-72
SRSF2	UBE2O	0.63906233	3.97E-72
SRSF1	DROSHA	0.638895875	4.44E-72
SRSF10	FANCL	0.63886874	4.52E-72
SRSF11	ZNF292	0.638656294	5.21E-72
SRSF10	ARL6IP6	0.638593577	5.43E-72
SRSF10	INO80D	0.63858989	5.44E-72
SRSF11	MAPK8IP3	0.638552538	5.58E-72
SRSF1	HNRNPH1	0.638529473	5.67E-72
SRSF11	ZMYM6	0.638501142	5.77E-72
SRSF11	PPP1R12A	0.638493841	5.80E-72
SRSF4	FBXO42	0.638357367	6.35E-72
SRSF7	FUS	0.638052429	7.78E-72
SRSF1	HMGB2	0.637973097	8.20E-72
SRSF2	STRADA	0.637936644	8.40E-72
SRSF10	TMX3	0.637933119	8.42E-72
SRSF1	ZNF639	0.637845423	8.92E-72
SRSF1	MSL2	0.637843993	8.93E-72
SRSF10	SMG1	0.637773488	9.36E-72
SRSF3	SUMO2	0.637702344	9.81E-72
SRSF1	RACGAP1	0.637623151	1.03E-71
SRSF1	NUP188	0.637618358	1.04E-71
SRSF1	SUZ12	0.637591923	1.06E-71
SRSF3	NOL7	0.637539187	1.09E-71
SRSF9	NIF3L1	0.637532031	1.10E-71
SRSF10	CBFB	0.63734638	1.24E-71
SRSF1	FNBP4	0.637299499	1.28E-71
SRSF9	C12orf49	0.637164198	1.40E-71
SRSF9	RCN2	0.637116004	1.45E-71
SRSF10	USP24	0.637066729	1.49E-71
SRSF10	ZNF638	0.637002616	1.56E-71
SRSF2	TADA2A	0.636950059	1.61E-71
SRSF1	NDC80	0.636796493	1.79E-71
SRSF9	RACGAP1	0.636682133	1.93E-71
SRSF9	HNRNPL	0.63660599	2.02E-71
SRSF11	WSB1	0.636551858	2.10E-71
SRSF10	TOPBP1	0.6365238	2.14E-71
SRSF2	HAUS5	0.636497786	2.17E-71
SRSF2	TTF2	0.636482801	2.20E-71
SRSF11	DYNC1LI2	0.636390015	2.33E-71
SRSF10	MBNL1	0.636355275	2.39E-71
SRSF3	SNRPB2	0.636305321	2.47E-71
SRSF1	RPIA	0.636284679	2.50E-71
SRSF2	NCBP2	0.636205873	2.64E-71
SRSF2	SUMO2	0.636053418	2.91E-71
SRSF2	ZNF248	0.635984368	3.05E-71
SRSF10	PRPF39	0.635917619	3.19E-71
SRSF11	ZC3H11A	0.63590116	3.22E-71
SRSF11	MED13L	0.635876857	3.27E-71
SRSF6	TCERG1	0.6358287	3.38E-71
SRSF10	APAF1	0.635758117	3.54E-71

SRSF1	RAD1	0.635753256	3.55E-71
SRSF2	XRCC3	0.635714243	3.64E-71
SRSF2	LY6G5B	0.635709271	3.66E-71
SRSF1	PHF12	0.635575985	3.99E-71
SRSF10	FBXO11	0.635543413	4.08E-71
SRSF1	CBFB	0.635473598	4.27E-71
SRSF2	NUP43	0.635469578	4.28E-71
SRSF11	CEP192	0.635402242	4.47E-71
SRSF1	UTP6	0.635398362	4.48E-71
SRSF2	RAD51D	0.635253888	4.93E-71
SRSF1	RALGAPB	0.635192277	5.13E-71
SRSF3	CLIC1	0.63511926	5.39E-71
SRSF1	CHAF1B	0.634969977	5.94E-71
SRSF11	FAM76B	0.634873722	6.33E-71
SRSF10	FGFR1OP2	0.6348308	6.51E-71
SRSF3	IQCB1	0.634802997	6.63E-71
SRSF10	CREB1	0.634780284	6.73E-71
SRSF10	GPD2	0.634768986	6.78E-71
SRSF9	TMED2	0.634743621	6.89E-71
SRSF1	MCM4	0.634609199	7.53E-71
SRSF9	USP39	0.63456132	7.77E-71
SRSF2	CPSF6	0.634540511	7.87E-71
SRSF1	FUS	0.634473644	8.22E-71
SRSF2	GINS1	0.634463337	8.28E-71
SRSF10	MAP4K5	0.634434913	8.44E-71
SRSF3	SET	0.634399922	8.63E-71
SRSF1	NAA25	0.63436105	8.85E-71
SRSF10	NEMF	0.634356709	8.88E-71
SRSF11	CCAR1	0.634350457	8.91E-71
SRSF2	PPHLN1	0.634297596	9.23E-71
SRSF1	HNRNPC	0.634275894	9.36E-71
SRSF3	MTF2	0.634239888	9.58E-71
SRSF10	CGGBP1	0.634227335	9.66E-71
SRSF9	HDAC2	0.634141955	1.02E-70
SRSF9	HMGN4	0.634107479	1.05E-70
SRSF7	SUPT7L	0.634013467	1.11E-70
SRSF2	RNF34	0.633930842	1.17E-70
SRSF1	SP1	0.633868142	1.22E-70
SRSF1	GPSM2	0.633706583	1.36E-70
SRSF2	CEP55	0.633637701	1.42E-70
SRSF1	UBE2N	0.633458583	1.60E-70
SRSF9	EIF2B1	0.633372229	1.69E-70
SRSF3	SNRPG	0.633245062	1.83E-70
SRSF10	TMF1	0.633208428	1.88E-70
SRSF9	SF3B2	0.63318599	1.91E-70
SRSF3	ZBTB12	0.633157518	1.94E-70
SRSF2	MASTL	0.633156658	1.94E-70
SRSF6	HNRNPH1	0.633154098	1.95E-70
SRSF10	PMS1	0.633025472	2.12E-70
SRSF11	SENP7	0.63295152	2.22E-70
SRSF1	SMURF2	0.632897146	2.30E-70

SRSF1	DTL	0.632831464	2.40E-70
SRSF9	RAD51	0.632785809	2.47E-70
SRSF1	NUP85	0.632738637	2.55E-70
SRSF1	KIF14	0.632721394	2.58E-70
SRSF3	HNRNPA2B1	0.632684598	2.64E-70
SRSF11	RBAK	0.632577834	2.83E-70
SRSF7	PRPF39	0.632572936	2.84E-70
SRSF10	CTDSPL2	0.632563793	2.86E-70
SRSF9	NCBP2	0.63251271	2.96E-70
SRSF1	SF3B1	0.632511468	2.96E-70
SRSF2	PTMA	0.632494378	2.99E-70
SRSF10	SPIN1	0.632490514	3.00E-70
SRSF11	IKZF4	0.632377062	3.23E-70
SRSF2	C4orf46	0.632376718	3.23E-70
SRSF6	PRPF38B	0.6323345	3.32E-70
SRSF10	USP1	0.632322886	3.34E-70
SRSF2	STIL	0.632240183	3.53E-70
SRSF10	CEP135	0.632148546	3.74E-70
SRSF11	ELF2	0.632029558	4.04E-70
SRSF9	KIF4A	0.632029334	4.05E-70
SRSF11	UBN2	0.631941209	4.28E-70
SRSF1	TAF4	0.631934963	4.30E-70
SRSF2	RAD51AP1	0.63192468	4.33E-70
SRSF6	CCNT2	0.631903113	4.39E-70
SRSF9	ARF3	0.631895736	4.41E-70
SRSF9	PSME3	0.63185191	4.54E-70
SRSF7	ACIN1	0.631842372	4.57E-70
SRSF10	SCAF8	0.631821055	4.63E-70
SRSF9	PSMD9	0.631722999	4.93E-70
SRSF4	PHACTR4	0.631711319	4.97E-70
SRSF3	NAP1L1	0.631545724	5.53E-70
SRSF3	SSB	0.631540652	5.55E-70
SRSF11	FBXO11	0.63147448	5.80E-70
SRSF1	RNF44	0.631390261	6.12E-70
SRSF9	EIF4A3	0.631326171	6.38E-70
SRSF1	EED	0.63129675	6.50E-70
SRSF9	PGAM1	0.631260419	6.66E-70
SRSF1	CCNF	0.63121154	6.87E-70
SRSF3	CBX1	0.631197283	6.93E-70
SRSF10	HNRNPH3	0.631162658	7.09E-70
SRSF9	YEATS2	0.631123021	7.28E-70
SRSF9	RNF41	0.631036344	7.70E-70
SRSF2	USP21	0.631032854	7.71E-70
SRSF1	RBM14	0.630946858	8.15E-70
SRSF1	CSNK1D	0.630864696	8.60E-70
SRSF2	SASS6	0.630802929	8.95E-70
SRSF11	SLTM	0.63073935	9.32E-70
SRSF2	CDCA8	0.630710802	9.50E-70
SRSF11	MAU2	0.630688737	9.63E-70
SRSF1	HNRNPA0	0.630454118	1.12E-69
SRSF2	KIF2C	0.63043743	1.13E-69

SRSF1	MAD2L1	0.630372526	1.18E-69
SRSF11	ATM	0.630316432	1.22E-69
SRSF2	DVL3	0.6302461	1.28E-69
SRSF7	HNRNPA3	0.630226703	1.30E-69
SRSF1	MZT1	0.630161484	1.35E-69
SRSF9	NONO	0.630122567	1.39E-69
SRSF11	OTUD3	0.630099006	1.41E-69
SRSF10	SOCS5	0.630053164	1.45E-69
SRSF11	TRA2A	0.630010108	1.49E-69
SRSF2	NUP188	0.629999314	1.50E-69
SRSF3	RAN	0.629968846	1.53E-69
SRSF1	RBM12	0.629926702	1.57E-69
SRSF1	CKAP2L	0.629840269	1.66E-69
SRSF11	ZFC3H1	0.629775928	1.73E-69
SRSF10	ZBTB11	0.629729414	1.79E-69
SRSF9	MAD2L1	0.629710484	1.81E-69
SRSF1	VEZF1	0.62970608	1.81E-69
SRSF10	MIS18BP1	0.629666668	1.86E-69
SRSF1	PALB2	0.629518689	2.05E-69
SRSF4	PPP1R8	0.629491229	2.08E-69
SRSF2	HSF2	0.62943875	2.16E-69
SRSF9	KIF23	0.6293626	2.26E-69
SRSF10	PHF12	0.629273994	2.40E-69
SRSF11	IQCB1	0.629252093	2.43E-69
SRSF1	PCNP	0.629251454	2.43E-69
SRSF2	C1orf52	0.629231486	2.46E-69
SRSF1	ATXN7L3	0.629205158	2.50E-69
SRSF2	GTSE1	0.629199344	2.51E-69
SRSF2	COIL	0.629116343	2.65E-69
SRSF1	RIF1	0.629109293	2.66E-69
SRSF1	PRPF40A	0.629094368	2.69E-69
SRSF9	LRRC59	0.628931397	2.98E-69
SRSF2	ZWINT	0.628803521	3.24E-69
SRSF10	FBXO5	0.628634239	3.61E-69
SRSF9	NUP205	0.628587831	3.72E-69
SRSF9	PGAM5	0.628450164	4.06E-69
SRSF10	ZNF567	0.628427674	4.12E-69
SRSF11	CEP290	0.628289047	4.50E-69
SRSF10	ZCCHC8	0.628248362	4.62E-69
SRSF10	TIA1	0.62821177	4.73E-69
SRSF10	METTL4	0.628199551	4.77E-69
SRSF1	DNAJC9	0.628132814	4.98E-69
SRSF3	TUBB	0.628080618	5.15E-69
SRSF2	DDX11	0.627934485	5.65E-69
SRSF10	CWC22	0.627872916	5.88E-69
SRSF3	WDHD1	0.627846061	5.98E-69
SRSF1	FOXM1	0.627790113	6.20E-69
SRSF1	STK4	0.627735994	6.42E-69
SRSF2	MMS22L	0.627691174	6.60E-69
SRSF10	MED13L	0.627615462	6.93E-69
SRSF1	ZMYM4	0.627611088	6.95E-69

SRSF3	NCBP2	0.627583095	7.07E-69
SRSF10	SENP6	0.627573463	7.12E-69
SRSF9	HAT1	0.627549808	7.23E-69
SRSF1	SPC25	0.627520763	7.36E-69
SRSF9	PIGS	0.627518149	7.37E-69
SRSF9	PPM1G	0.627512671	7.40E-69
SRSF2	FANCI	0.627407677	7.91E-69
SRSF11	ATAD2B	0.627330058	8.31E-69
SRSF11	CLK1	0.627324851	8.34E-69
SRSF11	ANKRD10	0.62731156	8.41E-69
SRSF11	ZBTB11	0.627282013	8.57E-69
SRSF3	RFC4	0.627262236	8.68E-69
SRSF1	C18orf54	0.627250517	8.75E-69
SRSF2	METTL3	0.627244735	8.78E-69
SRSF2	NAA25	0.627233033	8.84E-69
SRSF11	ZSCAN30	0.627223481	8.90E-69
SRSF10	SMARCAD1	0.627210326	8.97E-69
SRSF1	DNAJC10	0.627166926	9.22E-69
SRSF1	KIF20B	0.62706474	9.84E-69
SRSF11	ZNF224	0.626943512	1.06E-68
SRSF1	ENOPH1	0.626933765	1.07E-68
SRSF10	RERE	0.626858237	1.12E-68
SRSF2	IFT80	0.626839409	1.14E-68
SRSF1	C5orf34	0.626822708	1.15E-68
SRSF1	ZMYM1	0.626701034	1.24E-68
SRSF1	JRKL	0.626530556	1.38E-68
SRSF2	ORC1	0.626469466	1.44E-68
SRSF10	RNF34	0.626372384	1.53E-68
SRSF9	PRMT1	0.626299206	1.60E-68
SRSF11	ORC2	0.626229516	1.67E-68
SRSF11	CASP8AP2	0.626058565	1.87E-68
SRSF7	EWSR1	0.625810148	2.19E-68
SRSF1	TMEM237	0.625802097	2.20E-68
SRSF1	SUDS3	0.625762601	2.25E-68
SRSF1	ZNF169	0.625726744	2.30E-68
SRSF11	ZNF236	0.625700861	2.34E-68
SRSF2	FUS	0.625664653	2.40E-68
SRSF10	DMTF1	0.625476586	2.70E-68
SRSF3	SNRNP40	0.625475224	2.70E-68
SRSF1	SMPD4	0.625419939	2.80E-68
SRSF2	SLC25A19	0.625373313	2.88E-68
SRSF1	KIFC1	0.625366866	2.89E-68
SRSF2	KIF18A	0.625295329	3.03E-68
SRSF11	MSL2	0.625203671	3.21E-68
SRSF10	LEMD3	0.625141236	3.34E-68
SRSF3	RAE1	0.625107503	3.41E-68
SRSF1	CCDC93	0.625073615	3.48E-68
SRSF1	NPEPPS	0.625058857	3.52E-68
SRSF10	EP400	0.625048878	3.54E-68
SRSF2	UHRF1	0.62503978	3.56E-68
SRSF10	RSRC2	0.624998342	3.65E-68

SRSF1	SLBP	0.624917855	3.85E-68
SRSF7	THOC1	0.624901435	3.89E-68
SRSF9	PRC1	0.624899586	3.89E-68
SRSF10	TARDBP	0.624863661	3.98E-68
SRSF2	ASXL1	0.624711853	4.38E-68
SRSF11	SMG1	0.624640821	4.58E-68
SRSF1	PIP5K1A	0.624605994	4.68E-68
SRSF1	CENPL	0.624577207	4.77E-68
SRSF2	PRC1	0.624567148	4.80E-68
SRSF10	SCAF11	0.624427933	5.24E-68
SRSF9	INCENP	0.624412697	5.29E-68
SRSF11	CEP170	0.624362925	5.46E-68
SRSF9	MIS18A	0.624326957	5.58E-68
SRSF6	NCOA5	0.624315851	5.62E-68
SRSF1	OTUD3	0.624275334	5.77E-68
SRSF10	LUC7L3	0.624268464	5.79E-68
SRSF3	STMN1	0.624254325	5.85E-68
SRSF1	BPTF	0.62418529	6.11E-68
SRSF1	DUSP12	0.624150547	6.24E-68
SRSF2	USP36	0.624110443	6.40E-68
SRSF1	FAM72B	0.624101686	6.44E-68
SRSF7	NOP58	0.624100599	6.44E-68
SRSF10	XPO1	0.624098413	6.45E-68
SRSF1	TMEM39A	0.624063031	6.60E-68
SRSF10	ZNF146	0.624052314	6.64E-68
SRSF9	MAZ	0.624014013	6.80E-68
SRSF3	ANKLE2	0.623886814	7.37E-68
SRSF2	NUSAP1	0.623807331	7.75E-68
SRSF1	R3HDM1	0.623746149	8.05E-68
SRSF11	PRPF4B	0.623743806	8.06E-68
SRSF10	C4orf46	0.62374251	8.07E-68
SRSF9	PTGES3	0.623738826	8.09E-68
SRSF3	PRR3	0.623665271	8.47E-68
SRSF10	STIL	0.623656807	8.52E-68
SRSF6	SF3B1	0.623610246	8.77E-68
SRSF10	OTUD4	0.623557507	9.07E-68
SRSF3	FANCI	0.623455515	9.67E-68
SRSF10	MDM1	0.623445321	9.73E-68
SRSF9	DHX9	0.62343294	9.81E-68
SRSF3	RBM12	0.623377529	1.02E-67
SRSF10	ZNF292	0.623291352	1.07E-67
SRSF10	SUDS3	0.623251726	1.10E-67
SRSF3	MITD1	0.623211069	1.13E-67
SRSF1	CAPZA1	0.623174648	1.15E-67
SRSF9	SPPL3	0.623169242	1.16E-67
SRSF1	HAUS5	0.623132817	1.18E-67
SRSF3	ADO	0.623084294	1.22E-67
SRSF1	DNA2	0.623060781	1.24E-67
SRSF2	UBA2	0.62303967	1.26E-67
SRSF10	HACE1	0.622966623	1.31E-67
SRSF1	RPE	0.622941432	1.34E-67

SRSF3	NCL	0.622930876	1.34E-67
SRSF3	ILF3	0.622879962	1.39E-67
SRSF9	KCTD10	0.622821183	1.44E-67
SRSF9	RAD51AP1	0.622715338	1.54E-67
SRSF11	DIP2A	0.622700833	1.55E-67
SRSF2	ZNF26	0.622648507	1.60E-67
SRSF11	SP1	0.622596484	1.66E-67
SRSF1	PTBP2	0.62254026	1.72E-67
SRSF3	METTL9	0.622484831	1.78E-67
SRSF6	ACIN1	0.622412001	1.86E-67
SRSF3	RBM17	0.622362013	1.92E-67
SRSF1	RUNDC1	0.622309716	1.98E-67
SRSF1	LIG1	0.622300956	2.00E-67
SRSF1	SENP5	0.622282114	2.02E-67
SRSF2	GGNBP2	0.622254596	2.05E-67
SRSF1	HNRNPK	0.622236575	2.08E-67
SRSF1	SUV39H2	0.622195416	2.13E-67
SRSF3	WDR75	0.622180716	2.15E-67
SRSF3	KIF11	0.622090097	2.28E-67
SRSF1	SMNDC1	0.6220885	2.28E-67
SRSF2	ARHGAP11A	0.622000411	2.41E-67
SRSF2	TBP	0.621985581	2.43E-67
SRSF3	CCNB2	0.621920598	2.53E-67
SRSF3	KIF23	0.621919558	2.53E-67
SRSF1	USP1	0.621844198	2.66E-67
SRSF11	PHIP	0.621799657	2.73E-67
SRSF1	PDE7A	0.621794195	2.74E-67
SRSF1	DCAF7	0.621757471	2.80E-67
SRSF3	MTCH1	0.621707981	2.89E-67
SRSF9	SUV39H2	0.621628797	3.04E-67
SRSF2	MCM3	0.62162409	3.05E-67
SRSF10	RSBN1	0.621570247	3.15E-67
SRSF10	BRPF1	0.621562619	3.17E-67
SRSF1	ZRANB2	0.621560028	3.17E-67
SRSF11	HNRNPH3	0.621540399	3.21E-67
SRSF2	MELK	0.62148014	3.33E-67
SRSF11	PIP5K1A	0.621404198	3.50E-67
SRSF9	RAE1	0.621393072	3.52E-67
SRSF9	RFC4	0.621367028	3.58E-67
SRSF2	CENPF	0.621341317	3.64E-67
SRSF1	DDX52	0.621248933	3.85E-67
SRSF2	TTK	0.621245838	3.86E-67
SRSF1	LIN9	0.621229065	3.90E-67
SRSF2	MCM10	0.621227946	3.90E-67
SRSF10	TBK1	0.621223514	3.91E-67
SRSF3	TRIM59	0.621210136	3.95E-67
SRSF1	CAD	0.62119156	3.99E-67
SRSF11	SMCHD1	0.621049012	4.36E-67
SRSF1	DBF4	0.621017254	4.45E-67
SRSF3	TRIP4	0.620939479	4.67E-67
SRSF9	PCNP	0.620917839	4.73E-67

SRSF10	PIP5K1A	0.620905307	4.77E-67
SRSF11	UBXN4	0.62087376	4.87E-67
SRSF1	EP400	0.620837709	4.98E-67
SRSF9	DNMT1	0.620725992	5.33E-67
SRSF11	WDR47	0.62071694	5.36E-67
SRSF1	ZNF765	0.620712496	5.38E-67
SRSF10	ATR	0.620693121	5.44E-67
SRSF10	CBL	0.620684672	5.47E-67
SRSF2	ECT2	0.620630193	5.66E-67
SRSF1	CEP170	0.620469307	6.26E-67
SRSF3	EZH2	0.620454403	6.32E-67
SRSF1	DPF2	0.620437477	6.38E-67
SRSF9	GPN1	0.620425502	6.43E-67
SRSF2	KIF15	0.620409875	6.49E-67
SRSF9	LSM12	0.620395281	6.55E-67
SRSF10	ACTR2	0.620370096	6.66E-67
SRSF3	VRK1	0.620365073	6.68E-67
SRSF9	CDK2	0.620295525	6.97E-67
SRSF1	MAPRE1	0.620273156	7.07E-67
SRSF1	GGNBP2	0.620264595	7.11E-67
SRSF10	ELF2	0.62024631	7.19E-67
SRSF9	MCM4	0.620143003	7.67E-67
SRSF9	EFTUD2	0.620099716	7.87E-67
SRSF2	CHTF18	0.620039884	8.17E-67
SRSF9	EIF2S2	0.620035806	8.19E-67
SRSF1	CWC22	0.620023116	8.26E-67
SRSF11	ZNF567	0.619967013	8.55E-67
SRSF3	NCOA5	0.619937743	8.71E-67
SRSF11	ZNF131	0.619812863	9.41E-67
SRSF9	NUP93	0.619799987	9.49E-67
SRSF7	GTF3C2	0.619764075	9.70E-67
SRSF3	UTP6	0.619749229	9.79E-67
SRSF2	HNRNPU	0.619732403	9.89E-67
SRSF3	TMEM216	0.619726885	9.93E-67
SRSF11	ZBTB1	0.619657151	1.04E-66
SRSF10	HAUS6	0.619653087	1.04E-66
SRSF2	MTBP	0.61962403	1.06E-66
SRSF9	MCM10	0.619590513	1.08E-66
SRSF1	CNTRL	0.619408965	1.21E-66
SRSF10	CNTRL	0.619227595	1.35E-66
SRSF11	NEMF	0.619180942	1.39E-66
SRSF9	RRM1	0.619158369	1.41E-66
SRSF10	CTCF	0.619143809	1.42E-66
SRSF2	LIG1	0.619114759	1.45E-66
SRSF11	ZMYM2	0.619101318	1.46E-66
SRSF10	SASS6	0.619080363	1.48E-66
SRSF10	GIT2	0.618998802	1.56E-66
SRSF11	ILF3	0.618997929	1.56E-66
SRSF11	CEP95	0.618992049	1.56E-66
SRSF9	CKAP4	0.618945127	1.61E-66
SRSF6	REV1	0.618918555	1.64E-66

SRSF10	ZNF207	0.618869456	1.69E-66
SRSF1	CBFA2T2	0.618856351	1.70E-66
SRSF9	RUVBL1	0.618812615	1.75E-66
SRSF11	CDK17	0.618803343	1.76E-66
SRSF1	ZNF519	0.618780795	1.78E-66
SRSF10	MEX3C	0.618758195	1.81E-66
SRSF3	BTF3L4	0.61872574	1.85E-66
SRSF3	LSM2	0.618680646	1.90E-66
SRSF1	DLGAP5	0.61865117	1.93E-66
SRSF3	NRF1	0.61862883	1.96E-66
SRSF3	FBXO5	0.618555512	2.05E-66
SRSF1	EWSR1	0.618539845	2.07E-66
SRSF3	LMNB1	0.618474838	2.15E-66
SRSF2	WTAP	0.618473535	2.16E-66
SRSF1	CENPH	0.618468552	2.16E-66
SRSF1	RSRC2	0.61846	2.17E-66
SRSF7	TCERG1	0.618437824	2.20E-66
SRSF1	TADA2A	0.618392997	2.27E-66
SRSF3	TTF1	0.618377869	2.29E-66
SRSF10	C1orf109	0.618345892	2.33E-66
SRSF12	BEND3	0.618214086	2.53E-66
SRSF10	CEP85	0.618174284	2.59E-66
SRSF1	G2E3	0.618050571	2.80E-66
SRSF10	ATAD5	0.617904204	3.06E-66
SRSF9	UBE2Z	0.617862021	3.14E-66
SRSF11	AGAP6	0.61778763	3.29E-66
SRSF2	SPATS2	0.617760479	3.35E-66
SRSF10	PCNP	0.617749199	3.37E-66
SRSF1	UBE2Z	0.617748155	3.37E-66
SRSF11	ZC3H7A	0.617708572	3.46E-66
SRSF9	UBA2	0.617533749	3.85E-66
SRSF3	NFYA	0.617521434	3.88E-66
SRSF1	SMARCE1	0.617425226	4.11E-66
SRSF1	USP21	0.617348096	4.31E-66
SRSF9	MYBL2	0.617299945	4.44E-66
SRSF9	MCM3	0.617292611	4.46E-66
SRSF11	SCMH1	0.61728024	4.50E-66
SRSF2	CAD	0.617191698	4.75E-66
SRSF7	PTMA	0.617181124	4.78E-66
SRSF10	WDR47	0.617158847	4.85E-66
SRSF3	CENPO	0.61705418	5.17E-66
SRSF1	PRMT3	0.617042075	5.21E-66
SRSF7	SFPQ	0.61702934	5.25E-66
SRSF10	CLCN6	0.617028966	5.25E-66
SRSF9	RFC5	0.617016727	5.29E-66
SRSF1	STRN4	0.617005038	5.33E-66
SRSF9	CAND1	0.616906638	5.66E-66
SRSF3	CCDC43	0.616874526	5.77E-66
SRSF10	STXBP3	0.61675564	6.21E-66
SRSF10	CDK2	0.616706089	6.40E-66
SRSF3	PRPF38A	0.616641129	6.66E-66

SRSF1	ANAPC7	0.616535634	7.11E-66
SRSF7	SRRT	0.61650432	7.24E-66
SRSF3	KIF18A	0.616496881	7.28E-66
SRSF2	UBE2N	0.616474399	7.38E-66
SRSF11	PIKFYVE	0.616466324	7.41E-66
SRSF1	RNF219	0.616460719	7.44E-66
SRSF9	MSH2	0.616451736	7.48E-66
SRSF1	LRRC37B	0.616449902	7.49E-66
SRSF10	ZFC3H1	0.616409736	7.68E-66
SRSF1	SUPT16H	0.616394483	7.75E-66
SRSF10	ZNF131	0.616249088	8.47E-66
SRSF9	STIP1	0.616193015	8.77E-66
SRSF2	TOP2A	0.616162399	8.93E-66
SRSF1	PPP1R12A	0.616115193	9.19E-66
SRSF2	KIF20A	0.616106947	9.24E-66
SRSF1	MTA3	0.616065685	9.48E-66
SRSF9	CDCA8	0.616027206	9.70E-66
SRSF1	HNRNPR	0.616018545	9.76E-66
SRSF10	CHORDC1	0.616016969	9.76E-66
SRSF1	RIT1	0.615948849	1.02E-65
SRSF9	CCT7	0.615936682	1.03E-65
SRSF1	HNRNPUL1	0.615877027	1.06E-65
SRSF10	RNF138	0.615863489	1.07E-65
SRSF2	TRIP13	0.615851152	1.08E-65
SRSF1	CREB1	0.61579684	1.12E-65
SRSF1	NUP160	0.61575184	1.15E-65
SRSF1	HAUS6	0.615744643	1.15E-65
SRSF11	TDG	0.615699517	1.19E-65
SRSF3	PPHLN1	0.615678772	1.20E-65
SRSF1	CLSPN	0.615676747	1.20E-65
SRSF2	RFC4	0.615663406	1.21E-65
SRSF10	NAA25	0.615661232	1.21E-65
SRSF11	SPATA13	0.615651973	1.22E-65
SRSF2	QRICH1	0.61554486	1.30E-65
SRSF1	BAZ2A	0.615525441	1.32E-65
SRSF9	CALU	0.61543311	1.40E-65
SRSF9	UTP18	0.615430246	1.40E-65
SRSF10	VHL	0.615423408	1.40E-65
SRSF3	RPIA	0.615399713	1.42E-65
SRSF11	CHD6	0.615365405	1.45E-65
SRSF11	ANKRD36	0.615294292	1.52E-65
SRSF3	PBX2	0.615264119	1.55E-65
SRSF2	CCAR1	0.615233858	1.58E-65
SRSF11	SENP6	0.615204708	1.60E-65
SRSF10	MSL2	0.615131368	1.68E-65
SRSF1	UBE2T	0.615121703	1.69E-65
SRSF1	CLK2	0.615117791	1.69E-65
SRSF9	POLD1	0.615084601	1.73E-65
SRSF10	ECT2	0.615018038	1.80E-65
SRSF1	CDC45	0.61495771	1.87E-65
SRSF2	DNAJC9	0.614882305	1.95E-65

SRSF1	ASF1B	0.614831342	2.01E-65
SRSF1	CCDC66	0.614744272	2.12E-65
SRSF2	NDC80	0.614739105	2.13E-65
SRSF9	CCT8	0.614717968	2.16E-65
SRSF2	RPIA	0.614692133	2.19E-65
SRSF11	SCAF11	0.614661678	2.23E-65
SRSF1	DHX15	0.614655833	2.24E-65
SRSF2	XRCC2	0.61462848	2.28E-65
SRSF9	CEP55	0.614621757	2.29E-65
SRSF11	PAPOLG	0.614612757	2.30E-65
SRSF1	BCLAF1	0.61446155	2.52E-65
SRSF11	RAB2B	0.61445921	2.53E-65
SRSF1	UIMC1	0.614434244	2.57E-65
SRSF3	TARDBP	0.614430094	2.57E-65
SRSF2	GPSM2	0.614376661	2.66E-65
SRSF9	SSB	0.614357154	2.69E-65
SRSF3	RRP36	0.61434989	2.70E-65
SRSF11	RBM12B	0.614259187	2.85E-65
SRSF1	RBM17	0.614257593	2.86E-65
SRSF1	HCFC1	0.614219022	2.92E-65
SRSF2	ZNF639	0.61417363	3.01E-65
SRSF2	NASP	0.614163579	3.03E-65
SRSF3	PAK1IP1	0.614148077	3.05E-65
SRSF9	NEDD1	0.614108845	3.13E-65
SRSF10	MED23	0.614103418	3.14E-65
SRSF7	RBM5	0.614017023	3.31E-65
SRSF3	SSRP1	0.613992984	3.36E-65
SRSF10	CREBZF	0.613931918	3.48E-65
SRSF2	FOXM1	0.613923571	3.50E-65
SRSF10	CDK17	0.613748525	3.89E-65
SRSF9	NAP1L1	0.613747761	3.89E-65
SRSF1	CSTF3	0.613738216	3.92E-65
SRSF11	ZNF248	0.613722024	3.96E-65
SRSF3	GPN1	0.613714974	3.97E-65
SRSF4	PRPF38B	0.613684083	4.05E-65
SRSF10	QSER1	0.613658037	4.11E-65
SRSF9	CDK1	0.613607582	4.24E-65
SRSF9	ACLY	0.613605359	4.25E-65
SRSF11	RNPC3	0.613581881	4.31E-65
SRSF2	CENPO	0.613547167	4.40E-65
SRSF1	CCNB2	0.613541129	4.42E-65
SRSF1	PTPDC1	0.613504192	4.52E-65
SRSF3	DBF4	0.613471635	4.61E-65
SRSF7	HNRNPA2B1	0.613424778	4.74E-65
SRSF9	H2AFY	0.613408861	4.78E-65
SRSF3	CEP55	0.613356011	4.94E-65
SRSF1	UBAP2	0.613312965	5.07E-65
SRSF1	KIF4A	0.613305011	5.09E-65
SRSF1	GRPEL2	0.613304419	5.10E-65
SRSF9	MPZL1	0.613253622	5.26E-65
SRSF9	ILF2	0.613237946	5.31E-65

SRSF1	HSPA14	0.613236762	5.31E-65
SRSF3	STX6	0.61312516	5.68E-65
SRSF2	CASP2	0.613102694	5.76E-65
SRSF2	DENND4B	0.613088812	5.81E-65
SRSF1	PIF1	0.613082648	5.83E-65
SRSF1	CHEK1	0.613044483	5.97E-65
SRSF9	CENPO	0.613042855	5.97E-65
SRSF2	TROAP	0.612960205	6.28E-65
SRSF11	SUDS3	0.612919592	6.43E-65
SRSF2	SFSWAP	0.61291564	6.45E-65
SRSF3	EHMT2	0.61286999	6.63E-65
SRSF3	CENPQ	0.61283798	6.76E-65
SRSF9	BUB1	0.612763925	7.07E-65
SRSF3	XPO5	0.612683177	7.42E-65
SRSF1	PRDM4	0.612669594	7.49E-65
SRSF1	TPX2	0.612663422	7.51E-65
SRSF9	OIP5	0.612583598	7.89E-65
SRSF2	C5orf34	0.612491863	8.34E-65
SRSF1	DENR	0.612455466	8.52E-65
SRSF10	WDR82	0.612436893	8.62E-65
SRSF2	FAM104A	0.612404774	8.79E-65
SRSF1	KPNA2	0.6123958	8.83E-65
SRSF1	RNPS1	0.612382517	8.90E-65
SRSF3	TAF11	0.612333856	9.17E-65
SRSF10	ZBTB34	0.612321782	9.24E-65
SRSF11	MDM1	0.612310799	9.30E-65
SRSF1	GPATCH8	0.612281376	9.47E-65
SRSF7	IQCB1	0.612278246	9.48E-65
SRSF3	SNRPB	0.612270151	9.53E-65
SRSF9	RAB5C	0.612147053	1.03E-64
SRSF4	CDK11A	0.612145223	1.03E-64
SRSF9	TRA2B	0.612121374	1.04E-64
SRSF2	KIFC1	0.612076285	1.07E-64
SRSF3	HSPA14	0.612034084	1.10E-64
SRSF1	TROAP	0.61196885	1.14E-64
SRSF9	DDOST	0.611882467	1.20E-64
SRSF11	DNA2	0.611845782	1.23E-64
SRSF11	HECTD2	0.611833957	1.24E-64
SRSF10	ZNF484	0.611833116	1.24E-64
SRSF10	YEATS2	0.611813075	1.26E-64
SRSF9	CCNB2	0.611801973	1.26E-64
SRSF1	NUP205	0.611800967	1.26E-64
SRSF2	C17orf53	0.611777496	1.28E-64
SRSF7	TIA1	0.61175347	1.30E-64
SRSF1	POLD1	0.611727702	1.32E-64
SRSF10	PLK4	0.61171143	1.34E-64
SRSF1	MSL1	0.611626848	1.40E-64
SRSF2	NPHP3	0.611601572	1.43E-64
SRSF9	HNRNPU	0.61159241	1.43E-64
SRSF1	NUP62	0.61159165	1.43E-64
SRSF10	MASTL	0.61158141	1.44E-64

SRSF7	ADPGK	0.611569082	1.45E-64
SRSF2	METTL4	0.611527285	1.49E-64
SRSF10	ZDHHC21	0.611522931	1.50E-64
SRSF2	HDAC2	0.61147695	1.54E-64
SRSF10	COPB1	0.611459379	1.55E-64
SRSF3	GTPBP2	0.611440666	1.57E-64
SRSF2	ADPGK	0.611407866	1.60E-64
SRSF1	TCF3	0.611402219	1.61E-64
SRSF1	AEBP2	0.611394112	1.62E-64
SRSF10	SOCS4	0.611393263	1.62E-64
SRSF1	CENPJ	0.611373639	1.64E-64
SRSF1	ZNF638	0.611268641	1.74E-64
SRSF1	YTHDC1	0.611238624	1.77E-64
SRSF11	DDX20	0.611174528	1.84E-64
SRSF3	DHX9	0.611141973	1.88E-64
SRSF1	NCAPG2	0.611140605	1.88E-64
SRSF1	TACC3	0.611119712	1.91E-64
SRSF2	HGS	0.611103719	1.92E-64
SRSF10	CDC27	0.610958464	2.10E-64
SRSF1	MED12	0.610909389	2.16E-64
SRSF2	CHTOP	0.610866537	2.22E-64
SRSF9	DNAJC14	0.610825819	2.27E-64
SRSF9	ZNF207	0.610819065	2.28E-64
SRSF9	GTSE1	0.610817133	2.29E-64
SRSF6	NKTR	0.610815094	2.29E-64
SRSF3	H2AFY	0.610805541	2.30E-64
SRSF10	ARMC8	0.610724421	2.42E-64
SRSF9	YEATS4	0.610658631	2.52E-64
SRSF1	SET	0.610624673	2.57E-64
SRSF1	GTF3C3	0.610606652	2.59E-64
SRSF9	MCM6	0.610527735	2.72E-64
SRSF7	MTF2	0.610487591	2.79E-64
SRSF2	XPO1	0.610435362	2.88E-64
SRSF9	DBR1	0.610373539	2.98E-64
SRSF9	KIF18A	0.610361047	3.01E-64
SRSF9	YWHAB	0.610332565	3.06E-64
SRSF1	PHF21A	0.610324889	3.07E-64
SRSF10	DCLRE1B	0.610322522	3.08E-64
SRSF11	CPSF7	0.61029185	3.13E-64
SRSF9	ANLN	0.610240789	3.23E-64
SRSF2	SETD5	0.610107597	3.50E-64
SRSF9	BAK1	0.610099635	3.52E-64
SRSF2	CDC45	0.610079566	3.56E-64
SRSF10	SBNO1	0.610043766	3.64E-64
SRSF2	PTBP2	0.610031049	3.66E-64
SRSF10	GPATCH8	0.610018672	3.69E-64
SRSF3	HMGB2	0.610011554	3.71E-64
SRSF1	MTPAP	0.609974927	3.79E-64
SRSF1	HAUS1	0.609893059	3.98E-64
SRSF10	CASP2	0.609874383	4.03E-64
SRSF3	NUP107	0.609837508	4.12E-64

SRSF3	GMPS	0.609795176	4.22E-64
SRSF1	UBE2I	0.609781909	4.25E-64
SRSF2	MCM2	0.609773404	4.28E-64
SRSF10	NUP107	0.609709524	4.44E-64
SRSF9	ACTR6	0.609668076	4.55E-64
SRSF11	USP48	0.609659475	4.58E-64
SRSF1	SOCS7	0.60948556	5.08E-64
SRSF1	PDCL	0.609372558	5.44E-64
SRSF1	PTBP1	0.609347141	5.52E-64
SRSF1	LCORL	0.609335937	5.56E-64
SRSF9	GLRX3	0.609281567	5.74E-64
SRSF11	MKNK1	0.609275121	5.76E-64
SRSF2	KIAA1841	0.60921133	5.98E-64
SRSF9	SPATS2	0.609176671	6.11E-64
SRSF2	DNA2	0.609161454	6.17E-64
SRSF9	PLIN3	0.609114179	6.34E-64
SRSF10	RICTOR	0.609041821	6.62E-64
SRSF6	CREBZF	0.609018514	6.72E-64
SRSF1	RAD51	0.60901767	6.72E-64
SRSF3	BUB1B	0.60898798	6.84E-64
SRSF11	ADNP	0.608976398	6.89E-64
SRSF9	HAUS2	0.608972366	6.90E-64
SRSF2	CNOT6	0.608963904	6.94E-64
SRSF1	CENPI	0.608926385	7.09E-64
SRSF2	METTL17	0.608854003	7.41E-64
SRSF1	ZNF107	0.608761728	7.83E-64
SRSF3	ZCCHC3	0.608671821	8.26E-64
SRSF1	ATXN2L	0.608634969	8.44E-64
SRSF10	STX12	0.608588976	8.68E-64
SRSF2	DMTF1	0.60858097	8.72E-64
SRSF11	PHF2	0.608438747	9.49E-64
SRSF2	ZCCHC8	0.608353195	9.99E-64
SRSF1	SSB	0.608228479	1.08E-63
SRSF10	VEZT	0.608209459	1.09E-63
SRSF11	RC3H1	0.60818354	1.10E-63
SRSF10	ARID1B	0.608171837	1.11E-63
SRSF1	QRICH1	0.608119526	1.15E-63
SRSF1	ANKRD27	0.608072694	1.18E-63
SRSF11	SPEN	0.608006397	1.23E-63
SRSF1	FAM72A	0.607990389	1.24E-63
SRSF3	ABT1	0.607902535	1.31E-63
SRSF1	UBE2E1	0.607895672	1.31E-63
SRSF11	EP400	0.607801237	1.39E-63
SRSF10	DCK	0.60774154	1.44E-63
SRSF3	NUF2	0.607719355	1.46E-63
SRSF3	TJAP1	0.607691348	1.48E-63
SRSF1	E2F3	0.607679032	1.49E-63
SRSF1	TP53BP1	0.607670118	1.50E-63
SRSF9	M6PR	0.60765565	1.51E-63
SRSF9	KIF2C	0.607611007	1.55E-63
SRSF1	CEP135	0.607596832	1.57E-63

SRSF9	CCT6A	0.60757594	1.59E-63
SRSF9	DLGAP5	0.607566843	1.59E-63
SRSF9	ORC6	0.60751175	1.65E-63
SRSF11	KRIT1	0.60748657	1.67E-63
SRSF9	RBM12	0.607478796	1.68E-63
SRSF1	ASXL1	0.607456569	1.70E-63
SRSF10	UBXN4	0.60734778	1.82E-63
SRSF2	FAM72B	0.607321907	1.84E-63
SRSF11	CCDC82	0.607227856	1.95E-63
SRSF10	KNTC1	0.607182517	2.00E-63
SRSF9	PNO1	0.607166788	2.02E-63
SRSF9	DDX50	0.607144459	2.05E-63
SRSF2	CBFA2T2	0.60707632	2.13E-63
SRSF1	VHL	0.6070728	2.14E-63
SRSF2	CDK1	0.607059596	2.15E-63
SRSF9	CCNB1	0.607026034	2.20E-63
SRSF1	METTL3	0.60701058	2.22E-63
SRSF10	CENPE	0.607005618	2.22E-63
SRSF2	ACYP1	0.606943711	2.31E-63
SRSF3	UBE2I	0.606882078	2.39E-63
SRSF2	CENPE	0.606871539	2.41E-63
SRSF2	ZMYM1	0.606864183	2.42E-63
SRSF2	RSRC2	0.606841652	2.45E-63
SRSF11	SASS6	0.606840503	2.45E-63
SRSF10	RYK	0.606797271	2.52E-63
SRSF11	DYRK1A	0.606768455	2.56E-63
SRSF10	DIP2B	0.606748087	2.59E-63
SRSF7	ERCC3	0.606724126	2.63E-63
SRSF10	AZI2	0.606719004	2.64E-63
SRSF7	RSRC2	0.606695325	2.67E-63
SRSF2	SMURF2	0.606637052	2.77E-63
SRSF10	ZNF644	0.606623	2.79E-63
SRSF2	HSPA14	0.606606694	2.82E-63
SRSF2	CDC6	0.606525057	2.96E-63
SRSF6	TRA2A	0.606479076	3.04E-63
SRSF2	YEATS2	0.606390837	3.20E-63
SRSF1	DEPDC1	0.606362925	3.25E-63
SRSF2	LRRC37B	0.606340588	3.30E-63
SRSF1	C17orf80	0.606337293	3.30E-63
SRSF3	TPM3	0.606288657	3.40E-63
SRSF2	LMNB2	0.606253909	3.47E-63
SRSF1	BARD1	0.606253567	3.47E-63
SRSF11	EXTL2	0.606198989	3.58E-63
SRSF9	UBE2E1	0.606175158	3.64E-63
SRSF11	ZNF84	0.606158285	3.67E-63
SRSF10	CDC42	0.606130742	3.73E-63
SRSF10	KPNA6	0.606130347	3.73E-63
SRSF3	HMGB1	0.606099605	3.80E-63
SRSF2	ZBTB40	0.606096219	3.81E-63
SRSF11	LY6G5B	0.606081989	3.84E-63
SRSF9	PHF5A	0.606081614	3.84E-63

SRSF2	POLD1	0.606062381	3.89E-63
SRSF10	ZNF267	0.605981546	4.08E-63
SRSF3	NCAPH	0.605962985	4.12E-63
SRSF10	ATXN2L	0.605954357	4.14E-63
SRSF9	RBBP7	0.605904978	4.26E-63
SRSF2	UIMC1	0.605839702	4.43E-63
SRSF1	ANKRD52	0.60583744	4.44E-63
SRSF11	SGK494	0.605814601	4.50E-63
SRSF1	TET3	0.605813592	4.50E-63
SRSF10	MGA	0.605784238	4.58E-63
SRSF1	SHCBP1	0.605781123	4.59E-63
SRSF2	DBF4	0.605773924	4.61E-63
SRSF10	PRPF38A	0.605768021	4.62E-63
SRSF9	VPS26A	0.605765692	4.63E-63
SRSF2	HNRNPC	0.605756827	4.65E-63
SRSF1	VPS8	0.605753143	4.66E-63
SRSF10	NCBP1	0.605703141	4.80E-63
SRSF2	PLK1	0.605694773	4.83E-63
SRSF3	RCE1	0.605662028	4.92E-63
SRSF10	SMC5	0.605649566	4.96E-63
SRSF2	BRCA1	0.605638986	4.99E-63
SRSF1	ZCCHC3	0.605596561	5.12E-63
SRSF1	IARS	0.605561737	5.22E-63
SRSF10	TP53BP1	0.605497408	5.42E-63
SRSF11	TBC1D8	0.605448205	5.58E-63
SRSF1	MTA2	0.60541762	5.68E-63
SRSF9	PCBP2	0.605389964	5.78E-63
SRSF1	DCLRE1B	0.605306217	6.07E-63
SRSF1	UBAP2L	0.60529977	6.09E-63
SRSF2	TMEM39A	0.605258721	6.24E-63
SRSF11	PRDM10	0.605249766	6.28E-63
SRSF11	ZNF26	0.605243986	6.30E-63
SRSF10	TAOK1	0.605181991	6.53E-63
SRSF1	PURB	0.605167632	6.59E-63
SRSF1	RCN2	0.605156554	6.63E-63
SRSF6	RSRC2	0.605118304	6.78E-63
SRSF3	BTN2A1	0.605045854	7.08E-63
SRSF9	CDC123	0.604965621	7.42E-63
SRSF9	ZWILCH	0.604838527	7.99E-63
SRSF10	NDE1	0.604786505	8.24E-63
SRSF11	SCAF8	0.604785439	8.25E-63
SRSF2	ZNF169	0.604755679	8.39E-63
SRSF11	DDX17	0.604735663	8.49E-63
SRSF1	THUMPD2	0.604732819	8.51E-63
SRSF4	PANK4	0.60472733	8.53E-63
SRSF3	PRRC2A	0.604721286	8.57E-63
SRSF11	SLC30A7	0.604609225	9.15E-63
SRSF1	MEX3C	0.604600294	9.20E-63
SRSF9	H2AFZ	0.604598189	9.21E-63
SRSF1	POU2F1	0.604595577	9.22E-63
SRSF10	HAUS2	0.604560067	9.42E-63

SRSF1	CHD4	0.60453086	9.58E-63
SRSF2	ZNF202	0.604521504	9.63E-63
SRSF3	U2AF2	0.604504606	9.73E-63
SRSF10	INCENP	0.604472606	9.91E-63
SRSF1	SPIN1	0.604468708	9.94E-63
SRSF2	RBMX	0.60444206	1.01E-62
SRSF1	ZBED4	0.604440878	1.01E-62
SRSF11	ATXN7	0.6044149	1.03E-62
SRSF2	PIP5K1A	0.604403798	1.03E-62
SRSF3	MCM7	0.604393032	1.04E-62
SRSF2	ANAPC7	0.604346121	1.07E-62
SRSF10	PTBP2	0.604320579	1.08E-62
SRSF1	UTP20	0.604312869	1.09E-62
SRSF10	UBA3	0.60429268	1.10E-62
SRSF1	RBM45	0.604240211	1.14E-62
SRSF3	SNRPA1	0.604146925	1.20E-62
SRSF10	BUB1B	0.604135651	1.21E-62
SRSF3	RSL24D1	0.604130701	1.21E-62
SRSF11	SUPT7L	0.604100662	1.23E-62
SRSF2	LARP4B	0.604051717	1.27E-62
SRSF9	PSMD2	0.604049205	1.27E-62
SRSF2	MCM8	0.604035985	1.28E-62
SRSF3	DDX27	0.60403479	1.28E-62
SRSF1	ZNF567	0.604000436	1.31E-62
SRSF2	ZNF142	0.603990486	1.32E-62
SRSF10	HMGXB4	0.603960689	1.34E-62
SRSF7	HNRNPC	0.603916755	1.37E-62
SRSF3	CHEK1	0.603868229	1.41E-62
SRSF7	FANCL	0.603821084	1.45E-62
SRSF3	MORF4L1	0.60380798	1.46E-62
SRSF3	ANP32B	0.60379509	1.48E-62
SRSF2	KHDRBS1	0.603780916	1.49E-62
SRSF11	CCDC93	0.603748932	1.52E-62
SRSF10	SENP1	0.603746659	1.52E-62
SRSF10	PPP1CB	0.603740621	1.52E-62
SRSF3	KIF2C	0.603739148	1.52E-62
SRSF9	CENPA	0.603729833	1.53E-62
SRSF11	ZNF721	0.603707731	1.55E-62
SRSF3	DNAJC9	0.603680937	1.58E-62
SRSF1	ETAA1	0.603650499	1.61E-62
SRSF7	NUP107	0.60364499	1.61E-62
SRSF1	ZNF84	0.603638457	1.62E-62
SRSF10	TRIO	0.603561225	1.69E-62
SRSF1	NOL8	0.603436532	1.82E-62
SRSF9	KIF11	0.60343322	1.82E-62
SRSF6	MDM4	0.603425163	1.83E-62
SRSF1	C17orf53	0.603420472	1.84E-62
SRSF1	NAT10	0.603330698	1.94E-62
SRSF10	SLMAP	0.603317273	1.95E-62
SRSF10	NFATC2IP	0.60324517	2.04E-62
SRSF3	EXOSC3	0.603215559	2.07E-62

SRSF11	CCDC66	0.603197657	2.09E-62
SRSF10	PHIP	0.603138633	2.17E-62
SRSF2	PHF19	0.60313083	2.18E-62
SRSF10	ZNF326	0.603096039	2.22E-62
SRSF9	IFT52	0.602944664	2.43E-62
SRSF10	ARHGAP11A	0.60293292	2.44E-62
SRSF9	RPN2	0.60286675	2.54E-62
SRSF11	CAPZA1	0.602863007	2.54E-62
SRSF1	HECTD2	0.602812258	2.62E-62
SRSF10	REV1	0.602810035	2.62E-62
SRSF3	CD2AP	0.602794924	2.65E-62
SRSF1	PHF19	0.602772988	2.68E-62
SRSF1	PPP4R1	0.602763217	2.70E-62
SRSF10	SPIN4	0.602709393	2.78E-62
SRSF2	FANCB	0.602698573	2.80E-62
SRSF2	MCM6	0.602663501	2.86E-62
SRSF9	CDCA5	0.60266208	2.86E-62
SRSF1	DDX46	0.602589325	2.99E-62
SRSF1	ZBTB12	0.602445334	3.25E-62
SRSF10	RBM12	0.602428819	3.28E-62
SRSF6	PNISR	0.602425111	3.29E-62
SRSF3	MCM6	0.602424886	3.29E-62
SRSF11	PCF11	0.602375413	3.38E-62
SRSF10	ADNP	0.602348916	3.44E-62
SRSF10	CNOT6	0.602344521	3.44E-62
SRSF11	C1orf52	0.602331121	3.47E-62
SRSF11	HIPK1	0.602267267	3.60E-62
SRSF10	ZSCAN30	0.602253359	3.63E-62
SRSF10	NPAT	0.602250532	3.64E-62
SRSF1	ZDHHC17	0.602181029	3.79E-62
SRSF2	MSH2	0.602142253	3.88E-62
SRSF1	UBE2C	0.602120569	3.92E-62
SRSF10	ZNF248	0.602075123	4.03E-62
SRSF9	SLC25A19	0.602071476	4.04E-62
SRSF1	DDX20	0.602033243	4.13E-62
SRSF3	PCNP	0.601993851	4.22E-62
SRSF1	ACYP1	0.601957908	4.31E-62
SRSF3	SRRT	0.601917188	4.42E-62
SRSF9	HNRNPA3	0.601902109	4.46E-62
SRSF2	EXO1	0.601864009	4.56E-62
SRSF11	GON4L	0.601831567	4.64E-62
SRSF1	CGGBP1	0.601820519	4.67E-62
SRSF10	SGK494	0.601793073	4.75E-62
SRSF3	HMGN4	0.60176826	4.82E-62
SRSF2	ARMC8	0.601762292	4.83E-62
SRSF11	CCDC18	0.601733895	4.92E-62
SRSF1	UPF3B	0.60172723	4.93E-62
SRSF11	RASA2	0.601707816	4.99E-62
SRSF9	SBNO1	0.601658552	5.14E-62
SRSF2	MSL2	0.601615141	5.27E-62
SRSF2	ATXN2L	0.601607161	5.29E-62

SRSF3	DEPDC1B	0.601599718	5.31E-62
SRSF4	FOXJ3	0.601596874	5.32E-62
SRSF1	CDCA3	0.601594723	5.33E-62
SRSF10	RPAP3	0.601578308	5.38E-62
SRSF1	MAP4K5	0.601555467	5.45E-62
SRSF3	PTBP1	0.60154204	5.50E-62
SRSF2	BUB1	0.601541098	5.50E-62
SRSF2	PRPF3	0.601530516	5.53E-62
SRSF2	CENPJ	0.601510892	5.60E-62
SRSF10	TANK	0.601394757	5.99E-62
SRSF1	PDCD7	0.601394096	5.99E-62
SRSF1	CSNK2A1	0.601366926	6.08E-62
SRSF9	DPF2	0.601352561	6.13E-62
SRSF1	SOCS5	0.601318244	6.26E-62
SRSF3	LEMD2	0.601304405	6.31E-62
SRSF2	TRIM59	0.601303164	6.31E-62
SRSF11	ARID1B	0.601303051	6.31E-62
SRSF6	ZDHHC17	0.601296704	6.34E-62
SRSF1	PKMYT1	0.601296278	6.34E-62
SRSF9	PRIM2	0.6012618	6.47E-62
SRSF2	SKA3	0.601187482	6.75E-62
SRSF3	TRIM28	0.601104877	7.08E-62
SRSF9	GTF2H3	0.601103588	7.09E-62
SRSF3	TACC3	0.601069392	7.23E-62
SRSF2	NCOA5	0.601049569	7.32E-62
SRSF9	USP1	0.601007137	7.50E-62
SRSF3	HJURP	0.601002956	7.52E-62
SRSF3	C6orf48	0.600999278	7.53E-62
SRSF9	BTBD10	0.600967931	7.67E-62
SRSF1	FRMD8	0.600950752	7.75E-62
SRSF9	EIF4H	0.600925865	7.86E-62
SRSF10	BCOR	0.600902269	7.97E-62
SRSF7	ILF3	0.600878671	8.08E-62
SRSF1	EFCAB7	0.600847242	8.23E-62
SRSF1	RMI2	0.600810058	8.41E-62
SRSF1	SRRT	0.600775817	8.57E-62
SRSF1	WDR62	0.600757785	8.66E-62
SRSF3	RBM8A	0.600744587	8.73E-62
SRSF9	SENP1	0.600699613	8.96E-62
SRSF1	TAF1B	0.600633634	9.31E-62
SRSF9	CBX3	0.600547998	9.78E-62
SRSF2	STMN1	0.600520132	9.94E-62
SRSF3	PSPC1	0.600493305	1.01E-61
SRSF1	ZNF621	0.600486816	1.01E-61
SRSF1	FOXJ3	0.600461583	1.03E-61
SRSF6	ADNP	0.600446473	1.04E-61
SRSF1	NIF3L1	0.600428612	1.05E-61
SRSF9	LMNB1	0.600394548	1.07E-61
SRSF10	MAML1	0.600344908	1.10E-61
SRSF9	DKC1	0.600337742	1.11E-61
SRSF3	UBE2N	0.60026188	1.15E-61

SRSF9	TPX2	0.600216976	1.19E-61
SRSF10	ARID1A	0.600211903	1.19E-61
SRSF3	CPSF6	0.600197332	1.20E-61
SRSF2	ZDHHC17	0.600193448	1.20E-61
SRSF10	ZBTB1	0.600103184	1.27E-61
SRSF9	METTL9	0.600097561	1.27E-61
SRSF3	ECT2	0.600089781	1.28E-61
SRSF9	ACP1	0.600042427	1.31E-61
SRSF1	MIS18BP1	0.600002735	1.34E-61

Table S6. Univariate COX results of SRSF-related genes with $p < 0.05$.

Gene	Hazard Ratio	*p* Value	95% CI-Lower	95% CI-Upper
CENPA	1.573074233	1.03E-10	1.371123048	1.804770583
NCL	2.478288278	4.11E-10	1.8644148	3.294284505
CDCA8	1.574021477	4.41E-10	1.364952363	1.815113609
TRIP13	1.548860491	5.33E-10	1.34906317	1.778247952
KPNA2	1.59894401	6.05E-10	1.378106215	1.855170465
KIF2C	1.545187105	6.30E-10	1.346088756	1.773733847
PRR11	1.638649699	6.43E-10	1.401057717	1.916532633
GTPBP4	2.016354979	1.59E-09	1.605666769	2.532086657
MCM10	1.551470841	1.67E-09	1.344966594	1.789681455
CCT2	1.807202486	2.20E-09	1.488693032	2.193857803
PPM1G	2.271762364	2.28E-09	1.73578693	2.973236028
NOP58	2.371492922	3.46E-09	1.780768646	3.158174808
TPX2	1.464842739	3.98E-09	1.289955448	1.663440588
MYBL2	1.322137407	4.90E-09	1.204061403	1.451792506
ERCC6L	1.998746505	5.82E-09	1.583085701	2.523544737
TTK	1.572678447	6.62E-09	1.349560354	1.832683873
SPC25	1.562648964	6.67E-09	1.343812247	1.817122735
CCT5	1.859554164	7.10E-09	1.5072625	2.294186773
CCT4	2.220289124	8.80E-09	1.691921154	2.913660475
KIF18A	1.688972941	1.03E-08	1.411588808	2.020864418
ANLN	1.449862816	1.20E-08	1.275989542	1.647429008
KIF20A	1.472958367	1.45E-08	1.288321756	1.684056286
PLK1	1.513748921	1.50E-08	1.311353043	1.747382833
RAN	1.819893567	1.51E-08	1.479149179	2.239133579
CEP55	1.438200858	1.75E-08	1.267448705	1.631956939
DBF4	2.290355811	2.08E-08	1.714166071	3.060222595
DKC1	1.902197749	2.30E-08	1.518127196	2.383434197
DLGAP5	1.47230924	2.33E-08	1.285424198	1.686365093
SFPQ	2.33685136	3.57E-08	1.727951345	3.160317157
KIF4A	1.433259942	3.65E-08	1.2609202	1.629154694
NCAPG	1.507968192	3.73E-08	1.302738695	1.74552892
CDCA3	1.54506843	3.91E-08	1.32297418	1.804446746
HJURP	1.467510175	4.31E-08	1.279312272	1.683393618
CENPI	1.703653493	5.79E-08	1.40536282	2.065256874
NDC80	1.526482292	5.91E-08	1.31004036	1.778684276
CCT7	2.095777991	6.70E-08	1.602101411	2.741577628
PNO1	2.239671684	6.91E-08	1.670818396	3.002198961
BUB1	1.50802249	7.90E-08	1.298031612	1.751984935
PCBP2	2.381617677	8.40E-08	1.733881423	3.271332564

FBXO5	1.944066414	9.12E-08	1.523392766	2.48090598
DEPDC1	1.469996998	9.88E-08	1.275788308	1.693769382
NOP56	1.772742216	1.11E-07	1.434971771	2.190018666
GINS1	1.508903163	1.27E-07	1.295319639	1.757704189
CCNB1	1.426230838	1.48E-07	1.249343441	1.62816271
SKA3	1.529701186	1.62E-07	1.304766827	1.793412946
CCT6A	1.970858035	1.79E-07	1.52766663	2.542623708
BRIX1	1.937915719	1.88E-07	1.510964219	2.485510435
KIF23	1.466752967	2.27E-07	1.268696448	1.695728139
C18orf54	1.925708812	2.29E-07	1.502411603	2.468267965
CDK4	1.648089371	3.15E-07	1.360893465	1.995893612
FOXM1	1.449163183	3.21E-07	1.256983439	1.670725219
FAM72B	1.86279544	3.24E-07	1.467320341	2.364859776
EXO1	1.502435998	4.17E-07	1.28329355	1.759000448
GTSE1	1.503159109	4.56E-07	1.282997716	1.761100022
CBX3	1.970649449	4.59E-07	1.513913133	2.565179709
RUVBL1	1.864418894	5.02E-07	1.462279993	2.377149265
MCM6	1.517470779	5.13E-07	1.289509764	1.785731004
EZH2	1.621231909	5.21E-07	1.342421023	1.957949748
MCM2	1.391081143	5.39E-07	1.222626795	1.582745246
SSB	2.017728256	5.72E-07	1.532383888	2.656793345
KIAA1841	2.158280709	5.73E-07	1.596397647	2.917929392
STIP1	1.766981613	6.82E-07	1.411441557	2.212081688
YWHAQ	1.723540615	7.61E-07	1.38904344	2.138588445
CCNF	1.711245569	7.80E-07	1.382775459	2.117741805
HDAC2	1.951176213	7.86E-07	1.49655037	2.543909441
CAD	1.755224554	8.39E-07	1.403194249	2.195571452
FAM72A	1.961541323	8.40E-07	1.500288719	2.564602609
BUB1B	1.422240572	8.58E-07	1.236100388	1.636410978
NUF2	1.380016566	8.87E-07	1.213679012	1.56915107
RAD54L	1.45749638	9.04E-07	1.254061837	1.693932178
TMEM237	1.874581223	9.06E-07	1.458783011	2.408894767
DHX37	2.094908703	9.09E-07	1.55946543	2.814196705
LLPH	2.161196681	9.36E-07	1.58834738	2.940648343
RBM17	2.004218716	1.00E-06	1.51684982	2.648180862
KIFC1	1.370849689	1.06E-06	1.207764954	1.555955788
CDC45	1.408714843	1.08E-06	1.227450386	1.616747635
E2F6	2.185268828	1.17E-06	1.594410135	2.995088746
TUBA1C	1.47072437	1.20E-06	1.258689606	1.718477823
SUV39H2	1.991938134	1.23E-06	1.507796649	2.631533591
MELK	1.383728701	1.25E-06	1.213446554	1.577906427
NCAPH	1.493744393	1.28E-06	1.269857752	1.757104138
CENPE	1.647133644	1.34E-06	1.345404715	2.016530201
UBE2C	1.298238109	1.35E-06	1.167827007	1.443212203
HAT1	1.915903697	1.42E-06	1.47094113	2.495468311
CDC6	1.376433524	1.47E-06	1.208593404	1.567581984
RBM45	3.067383947	1.48E-06	1.94351454	4.841149413
BEND3	1.971698428	1.53E-06	1.494932743	2.600514779
PGAM5	2.010205277	1.57E-06	1.511726913	2.673052401
KIF18B	1.397104403	1.75E-06	1.218107145	1.602404781
CDK1	1.361202783	1.88E-06	1.199093922	1.545227595

CDCA5	1.404391555	1.93E-06	1.221138434	1.615145003
CENPO	1.671716786	1.97E-06	1.352715895	2.065945274
LMNB2	1.507519092	2.10E-06	1.27235479	1.786147882
DENR	2.058794832	2.18E-06	1.52694509	2.775892982
SNRPD1	1.63523961	2.19E-06	1.334072894	2.004394659
YWHAB	2.021880332	2.24E-06	1.510350504	2.706656546
H2AFZ	1.511518738	2.40E-06	1.273101074	1.794585631
LMNB1	1.449080818	2.50E-06	1.241734304	1.69105034
ACP1	2.204735992	2.51E-06	1.586330951	3.06421607
CORO1C	1.691826819	2.75E-06	1.358014612	2.107693071
NEK2	1.364642532	2.83E-06	1.19814301	1.554279602
CLSPN	1.457511561	2.95E-06	1.244537778	1.706930869
ECT2	1.402413008	3.01E-06	1.216850089	1.616273248
TROAP	1.37520375	3.07E-06	1.20293788	1.572138832
MAD2L1	1.452227513	3.14E-06	1.241380904	1.69888609
CHORDC1	1.763334021	3.20E-06	1.388913434	2.238690184
PAK1IP1	1.799480306	3.30E-06	1.404861482	2.304945657
EIF2S2	1.831555295	3.31E-06	1.4192541	2.363632277
WDHD1	1.727739691	3.38E-06	1.371840385	2.175970669
POLQ	1.659450613	3.50E-06	1.339773287	2.055404721
ORC6	1.483458147	3.94E-06	1.254659894	1.753979772
PTMA	1.794358517	3.96E-06	1.399724686	2.300254129
HNRNPM	2.149882448	4.00E-06	1.552843796	2.97647101
ORC1	1.546978054	4.11E-06	1.28485381	1.862578514
SSRP1	1.850102799	4.34E-06	1.423036872	2.405334979
ANP32B	1.699735241	4.34E-06	1.355500605	2.131389598
CDCA4	1.61245562	4.50E-06	1.314710877	1.977631107
CKAP2L	1.4196293	4.97E-06	1.221391733	1.650041746
NCBP2	1.879997123	5.26E-06	1.432742474	2.46686983
NCAPD2	1.543278637	5.28E-06	1.280361347	1.860184985
PTGES3	2.052230647	5.29E-06	1.505974448	2.796628212
RAD51	1.489396042	5.40E-06	1.254473823	1.768311567
GMPS	1.838473158	5.57E-06	1.413654202	2.390954979
GRPEL2	2.044046968	6.04E-06	1.499697861	2.785979841
NCAPG2	1.581370927	6.61E-06	1.295564738	1.930226976
TACC3	1.433128893	6.62E-06	1.225461055	1.675988328
ZWINT	1.352545706	6.70E-06	1.185951793	1.542541525
GPSM2	1.781459143	6.98E-06	1.384873714	2.291614497
FANCB	1.796972455	7.07E-06	1.3914436	2.320690543
YTHDF2	2.358884317	7.83E-06	1.619129741	3.43662097
STMN1	1.35978175	7.97E-06	1.188211502	1.55612566
MKI67	1.353075767	8.17E-06	1.184734199	1.54533737
BTF3L4	2.002437392	8.26E-06	1.475657515	2.717267027
RFC4	1.490205598	8.50E-06	1.250197235	1.776289902
CHEK1	1.611910342	8.67E-06	1.306102837	1.989318817
SNRNP40	1.8763053	9.06E-06	1.42108117	2.477354322
RPIA	1.800539961	9.24E-06	1.388401671	2.335018908
SHCBP1	1.428794299	9.27E-06	1.22028647	1.67292943
NAP1L1	1.554069685	9.83E-06	1.278157422	1.889542354
EFTUD2	1.841866578	9.86E-06	1.404876883	2.41478277
POLA1	1.903048119	1.01E-05	1.430181546	2.532260436

FANCL	1.789779637	1.03E-05	1.381793931	2.318226383
RACGAP1	1.448833204	1.10E-05	1.228080787	1.709266747
VRK2	1.882664161	1.16E-05	1.418955384	2.497911055
SET	1.796314618	1.34E-05	1.379938566	2.338325984
NUP107	1.724492559	1.38E-05	1.348857078	2.204736614
MAPKAPK5	2.246984461	1.41E-05	1.559180141	3.238201305
DEPDC1B	1.339751717	1.50E-05	1.173575316	1.529458434
PPP1CB	1.881315337	1.53E-05	1.412706237	2.505366866
CENPL	1.670984299	1.59E-05	1.323470425	2.10974758
UPF3B	1.749035811	1.60E-05	1.356780582	2.254694907
SNRPB2	1.878710975	1.63E-05	1.410331245	2.502642511
MASTL	1.735191322	1.65E-05	1.350377164	2.229665168
PPP1CC	1.857004124	1.70E-05	1.400591312	2.46214887
ASF1B	1.340273866	1.72E-05	1.172737321	1.53174458
RAD51AP1	1.428078838	1.72E-05	1.213894831	1.680054248
CALU	1.502392307	1.74E-05	1.247780097	1.808958685
CENPH	1.578494764	1.76E-05	1.281588778	1.944185033
DDX27	1.877398093	1.83E-05	1.407452649	2.504257321
FANCE	1.62337913	1.86E-05	1.300401428	2.02657406
PRC1	1.346296422	1.92E-05	1.174676172	1.542990399
UTP18	1.745615677	1.94E-05	1.351851376	2.254074778
MCM4	1.393482221	1.96E-05	1.196575752	1.622791283
GPD2	1.6595605	1.98E-05	1.315025825	2.094362712
TPM3	1.666203218	2.00E-05	1.317754462	2.106790943
SLC25A19	1.612171287	2.03E-05	1.294262247	2.008168178
KIF15	1.48193367	2.06E-05	1.236529535	1.776041202
NUP205	1.775678256	2.09E-05	1.363034588	2.313245237
MSH2	1.58906254	2.19E-05	1.283130018	1.96793756
PPHLN1	2.259094042	2.25E-05	1.549762398	3.29308925
LCORL	1.915620819	2.26E-05	1.418183742	2.587537155
CENPF	1.356136826	2.55E-05	1.176821428	1.562774986
TOP2A	1.255894849	2.65E-05	1.129264163	1.39672534
UTP6	2.002969995	2.66E-05	1.448545873	2.769597344
POLD1	1.616743357	2.68E-05	1.29200031	2.023110258
WDR75	1.967310654	2.72E-05	1.434175462	2.698631593
CCT8	1.861747135	2.76E-05	1.39231277	2.48945673
DCTN2	1.764447033	2.76E-05	1.35307537	2.300886853
PHF19	1.47501594	2.78E-05	1.229848742	1.76905659
CDK2	1.588905226	2.80E-05	1.279392355	1.973296
LRRC59	1.627567098	2.84E-05	1.295686417	2.044456609
GPN1	1.940910673	2.85E-05	1.42274129	2.647799897
HSPA14	1.887458222	2.86E-05	1.401669176	2.541611532
KIF11	1.464057332	2.87E-05	1.224613853	1.750318164
UBE2E1	1.83058028	2.88E-05	1.378906191	2.430204595
CLIC1	1.340874769	2.89E-05	1.168652187	1.538477544
BRCA1	1.52763574	2.91E-05	1.252401857	1.863356351
RAD1	1.944012467	2.97E-05	1.422991164	2.655803189
GATC	2.22615052	2.98E-05	1.528992758	3.241183525
NAT10	2.070415207	3.01E-05	1.470983154	2.914118438
CDC123	1.683156172	3.07E-05	1.317618673	2.150102118
NONO	1.717970413	3.21E-05	1.331177053	2.217152355

XRCC2	1.554500404	3.24E-05	1.262526612	1.913996491
CHAF1B	1.452295465	3.38E-05	1.217460916	1.732426964
RBM14	2.172313398	3.50E-05	1.504374696	3.136815256
MPZL1	1.464943621	3.53E-05	1.222502442	1.755464642
EEF1E1	1.523448168	3.60E-05	1.247663908	1.86019192
MCM8	1.5805985	3.87E-05	1.270937244	1.965708086
CDC42	1.812379913	3.92E-05	1.365112213	2.406191166
ARHGAP11A	1.463987098	4.09E-05	1.220254151	1.756403141
VPS26A	1.844528544	4.44E-05	1.374867315	2.474628287
UBAP2	1.82774215	4.49E-05	1.368101397	2.441808314
SNRPG	1.71529401	4.57E-05	1.323322327	2.223368775
BAK1	1.401569674	4.66E-05	1.191364497	1.648863597
UHRF1	1.318839823	4.76E-05	1.15417591	1.506995998
PSMD2	1.695405059	4.84E-05	1.314255264	2.187092868
OIP5	1.384339464	4.86E-05	1.183306622	1.619525924
NUP85	1.736330808	5.00E-05	1.329934521	2.266912113
LSM12	1.896778994	5.00E-05	1.392067033	2.584480823
EIF4A3	1.701583657	5.03E-05	1.315985491	2.20016631
CKAP5	1.791807688	5.06E-05	1.351435898	2.375676712
SNRPB	1.480455391	5.12E-05	1.224426847	1.790019691
PIF1	1.524586197	5.19E-05	1.242940305	1.870052056
KHDRBS1	1.900187408	5.26E-05	1.392088649	2.593737252
CCDC59	2.112271407	5.28E-05	1.46996986	3.035225835
FANCI	1.483226954	5.51E-05	1.224615369	1.796451565
RPE	1.909017822	5.56E-05	1.394029536	2.614255259
SPATS2	1.617022217	5.76E-05	1.279434382	2.043684995
RAE1	1.805838408	5.78E-05	1.353913438	2.408612149
UBE2T	1.330144465	5.81E-05	1.157440316	1.528618171
DDOST	1.591702702	5.83E-05	1.268925827	1.996584384
USP39	1.973590581	6.29E-05	1.414611052	2.753449279
PSME3	1.678740582	6.54E-05	1.30176797	2.164878845
LARP4B	1.778428916	6.67E-05	1.340112485	2.360107412
CNOT6	1.817539804	6.82E-05	1.354501235	2.4388689
FANCD2	1.52805004	6.83E-05	1.240233627	1.882658939
PRIM2	1.74355903	6.84E-05	1.326152486	2.292344301
CCNB2	1.316220791	6.89E-05	1.149652469	1.506922497
NUP37	1.638198805	7.08E-05	1.284194853	2.089788258
NUP62	1.678366361	7.09E-05	1.300034756	2.166798718
ANAPC7	1.813203219	7.22E-05	1.351516417	2.432605235
GTF3C2	1.820618479	7.75E-05	1.352599952	2.450577971
CPSF6	1.773807881	8.02E-05	1.334152844	2.358346282
ACTL6A	1.606764502	8.14E-05	1.269138517	2.034208347
CDC7	1.450764067	8.22E-05	1.205501005	1.745926689
CENPK	1.453196326	8.37E-05	1.206267095	1.750673271
RBL1	1.709015387	8.45E-05	1.308304662	2.232456765
RBBP4	1.64388928	8.71E-05	1.282469307	2.107163074
IARS	1.586607174	9.04E-05	1.25923609	1.999086861
ZNF207	1.925320275	9.21E-05	1.386469728	2.673594733
NUP43	1.785052695	9.57E-05	1.33421362	2.388233097
TRIM28	1.53192856	0.000102805	1.235263169	1.899842212
XPO5	1.651378483	0.000103208	1.281991908	2.12719821

MMS22L	1.969953002	0.000103934	1.398812645	2.774292071
PHF5A	1.765477707	0.000107641	1.324114397	2.353959402
C5orf34	1.662898953	0.00010873	1.285331165	2.151377793
RPN2	1.601111184	0.000110215	1.261275154	2.032512109
FOXJ3	1.8292112	0.000110649	1.346795576	2.484425754
C4orf46	1.798434208	0.000110865	1.335526481	2.421790693
MTBP	1.797753218	0.000111645	1.33510134	2.420727579
MCM7	1.383424182	0.00011199	1.173382417	1.631064553
FOXK2	1.644286901	0.000113336	1.277365921	2.116605247
ARPC2	1.58612348	0.000117641	1.254224783	2.005850729
SMARCD1	1.649946908	0.000124103	1.277645243	2.130736067
WDR62	1.497196956	0.000127971	1.217837831	1.84063811
SNRPA1	1.590732504	0.000129344	1.254209217	2.017550075
HNRNPA3	1.787356995	0.000130231	1.32742659	2.406645347
DCLRE1B	1.791362682	0.000130602	1.328804009	2.414938725
SAE1	1.514253865	0.000130907	1.224213441	1.873010613
PSMD9	1.88247636	0.000131118	1.361230651	2.603318728
DDX11	1.450191193	0.00013263	1.198489578	1.754754096
PRPF38A	1.863268596	0.00013501	1.353625654	2.564793191
RMI2	1.285910064	0.000138023	1.129938037	1.463411832
PDIA6	1.499262881	0.000142447	1.216903308	1.847138692
INCENP	1.536044335	0.000143378	1.231169184	1.9164159
KPNB1	1.634389398	0.000144125	1.268642877	2.105579713
SASS6	1.765555596	0.000145364	1.316760165	2.367315358
DTL	1.321427689	0.00014922	1.144157816	1.526162835
TARDBP	1.715768749	0.000149996	1.2979111	2.268154113
COMMD2	1.759879427	0.000153281	1.3133765	2.358178024
RCC2	1.408202645	0.000153757	1.179454449	1.681315197
MTA3	1.696349431	0.000158354	1.289536408	2.231500697
MZT1	1.558383079	0.000159594	1.237814807	1.961971861
ILF2	1.54689612	0.000165619	1.23273318	1.941123713
ACYP1	1.521800023	0.000169244	1.222715876	1.894042072
H2AFY	1.6112851	0.0001703	1.256504179	2.066240381
HAUS1	1.585676883	0.000175339	1.246322571	2.017432112
NUP93	1.657168582	0.000177053	1.272634403	2.157892088
KIF14	1.498832089	0.000177576	1.21302213	1.851984043
AACS	1.472922253	0.000178681	1.202868982	1.803604546
CEP85	1.58059694	0.000190411	1.24276259	2.010268662
RPAP3	2.071335989	0.000192441	1.412594325	3.037271709
DDX55	1.827941256	0.000200024	1.330160403	2.512004738
PKMYT1	1.333616702	0.000200067	1.145873688	1.552120034
CKAP4	1.347535053	0.000208018	1.151033608	1.577582709
MAPRE1	1.446955317	0.00021304	1.189949086	1.759469977
EIF2B1	2.009815416	0.000228105	1.386576273	2.913188468
TAF1B	1.818440343	0.000236231	1.322090153	2.501134491
DBF4B	1.689047874	0.000251319	1.275756934	2.236227485
RRM1	1.480754035	0.000254512	1.199857662	1.827410519
UTP20	1.867969107	0.000259625	1.335836347	2.612077888
HNRNPL	1.943808536	0.000263535	1.360190137	2.777840774
CBX1	1.480904983	0.0002662	1.199116537	1.828912787
NEDD1	1.723923323	0.000269202	1.2861114	2.310773098

RNF34	1.710607804	0.000272004	1.281241161	2.283862825
CENPQ	1.503881012	0.00027787	1.206879818	1.873971264
SLBP	1.600636624	0.000284097	1.241564396	2.063555956
CSNK1D	1.711550559	0.000292774	1.279624513	2.289269458
STIL	1.559856764	0.000292826	1.226277357	1.984178466
HSF2	1.715860078	0.000293771	1.281010929	2.298322162
UBE2N	1.89617446	0.000312163	1.339063762	2.685068243
MCM3	1.345258797	0.000314061	1.144843208	1.580759023
YEATS2	1.606698796	0.000326027	1.240565809	2.080890026
CCDC43	1.680256731	0.000338209	1.265114783	2.231625715
USP1	1.520277563	0.000352906	1.208174132	1.91300559
MTCH1	1.620527935	0.000354012	1.243436422	2.111978337
PLIN3	1.421685217	0.00035643	1.171985959	1.724584533
ARL6IP6	1.579831771	0.000360292	1.228846592	2.031065913
C17orf53	1.516402715	0.00036714	1.205981468	1.906726806
DR1	1.617294511	0.000368389	1.24135625	2.107083712
PIGS	1.432694999	0.000370937	1.175389041	1.746328142
RAB35	1.733399176	0.000382579	1.279586332	2.348159424
GLRX3	1.614525158	0.000390344	1.238997098	2.103872149
ACTR6	1.762510497	0.000394796	1.288231638	2.411401148
SPPL3	1.733869579	0.000401488	1.278346457	2.351712794
UBE2Z	1.555320663	0.000402657	1.217773651	1.986430206
FUBP1	1.517026596	0.000403841	1.204245478	1.911046986
TRA2B	1.994401892	0.000405639	1.360342283	2.923998582
C1orf52	1.854588988	0.000407851	1.316791323	2.612031424
PLK4	1.452680516	0.000412521	1.180803959	1.787155831
HAUS2	1.830927482	0.000414126	1.308777627	2.561394216
HNRNPR	1.637008783	0.000424225	1.244569318	2.153192849
CASP2	1.591242945	0.000433706	1.228473381	2.061138768
VRK1	1.535772977	0.000436315	1.209184727	1.950569324
RNPS1	1.60413603	0.000452134	1.231850299	2.088932726
QSER1	1.754562348	0.00047029	1.280337994	2.404434647
RIT1	1.511957353	0.000473034	1.199146102	1.906369068
PDCD10	1.617607023	0.000485751	1.234583504	2.119461723
NASP	1.462473614	0.000493618	1.180921707	1.811152304
NXT1	1.526098685	0.000497085	1.202974356	1.936015662
BARD1	1.609287183	0.000538822	1.229153199	2.10698328
HMGB2	1.314434967	0.00054142	1.125811489	1.534661263
GRB2	1.629628141	0.000554144	1.235118714	2.150147875
ENOPH1	1.602627172	0.00055892	1.226007529	2.094941338
RBM12	1.738178311	0.00056406	1.269490457	2.379902761
RAD18	1.837000755	0.0005835	1.298957345	2.597908073
TUBA1B	1.296649216	0.000585104	1.118179923	1.503603448
SRRT	1.618858833	0.00064612	1.227479073	2.135029409
CDC27	1.686252292	0.000651959	1.248696879	2.277131337
HNRNPU	1.723069135	0.000683551	1.258713641	2.358731283
PPP1R8	1.792295135	0.000710544	1.278505089	2.512560865
HELLS	1.389939978	0.000716697	1.148558275	1.682050606
DDX23	1.783395274	0.000725535	1.275103239	2.494306818
ZMYM4	1.710069633	0.000727271	1.252735337	2.334362305
PWP1	1.90783988	0.000730355	1.311486363	2.77536474

CAND1	1.675195755	0.000756332	1.240752417	2.261757285
DDX54	1.739898943	0.000783045	1.259410837	2.403701987
U2AF2	1.713711352	0.000785172	1.251393906	2.346828271
CAPZA1	1.591496774	0.00080296	1.212827231	2.088394716
PCNP	1.703083067	0.000838167	1.246056929	2.327736288
C1orf109	1.785137987	0.000872035	1.269090124	2.511025477
LSM2	1.408904332	0.000924962	1.150238225	1.725739395
NUP188	1.585824868	0.000954435	1.206280871	2.084788519
NUSAP1	1.255672629	0.00099075	1.096550344	1.437885419
TTF2	1.586279723	0.001011542	1.204782407	2.088579102
TOPBP1	1.52161607	0.001011718	1.184701292	1.954345353
AEBP2	1.760057073	0.001013648	1.256353363	2.465708288
ZMYM1	1.709336023	0.001031587	1.241012993	2.354390854
KNTC1	1.372626047	0.00103167	1.136060849	1.658451892
GTF2H3	1.547436761	0.00105348	1.191697023	2.009370237
UBA2	1.535664152	0.001063235	1.187804318	1.98539806
RBBP7	1.566265144	0.001182886	1.194246881	2.054170325
SPIN4	1.518247708	0.001219694	1.178821323	1.955407541
SMC4	1.334611518	0.001226313	1.12034761	1.589852905
PRMT3	1.686399667	0.001257176	1.227586684	2.316694921
GTF3C3	1.86527727	0.001328043	1.274749865	2.729366278
RRP36	1.544258596	0.001379785	1.183285278	2.015350531
HAUS6	1.60545771	0.001384974	1.201112909	2.145921869
ANKLE2	1.576524889	0.001451462	1.191272454	2.086366319
ATAD5	1.632952136	0.001477298	1.20690286	2.209401241
DCK	1.417759119	0.001482162	1.143166212	1.758310296
PRKAG1	1.811720574	0.001486794	1.255686746	2.613973149
ZNF131	1.682553746	0.00151036	1.220039701	2.320405725
RAD51D	1.729042299	0.001575667	1.231192899	2.428203798
SENP1	1.79508646	0.001644455	1.247054579	2.583956994
ZNF639	1.785048741	0.00167065	1.243758369	2.561911613
DNMT1	1.375530958	0.00169706	1.1272029	1.678566846
SF3B2	1.760069799	0.001723766	1.235901778	2.50654684
SMNDC1	1.699606992	0.001735737	1.219559715	2.368612123
C12orf49	1.321169305	0.001762288	1.109578991	1.573108671
CENPJ	1.608217794	0.001765528	1.194025934	2.16608735
DDX52	1.691004175	0.001777111	1.216359864	2.35086277
FANCG	1.418897894	0.001835129	1.138582418	1.768226175
RIOK1	1.523917981	0.001902179	1.168085375	1.988147496
PTPDC1	1.626057308	0.001902963	1.196354162	2.210100031
XRCC3	1.55460382	0.001918665	1.176435403	2.054335523
TLK2	1.809425449	0.001980899	1.242645591	2.634717797
FBXO30	1.669219072	0.001981287	1.206459466	2.309478593
DDX50	1.638612363	0.001985362	1.198234226	2.24083941
POU2F1	1.900770697	0.001985728	1.26514883	2.855734562
RCN2	1.371462873	0.001989976	1.122581182	1.675522841
TRIM59	1.542969278	0.001993007	1.172014103	2.031335789
SUPT7L	1.715658927	0.001995482	1.218362808	2.415935167
HNRNPK	1.822565995	0.001997051	1.245574439	2.666839255
MTF2	1.583894057	0.002011277	1.182993489	2.120654431
CD2AP	1.435250443	0.002097259	1.140092049	1.806822384

POGK	1.414043738	0.002138288	1.13347954	1.764054509
NIF3L1	1.667365419	0.002146277	1.202940706	2.311092665
SCMH1	1.437229582	0.002156315	1.139950153	1.812034382
PTBP1	1.685567093	0.002183962	1.206986632	2.353908777
UBE2I	1.497612346	0.002294769	1.155194206	1.94152873
RBMX	1.584227889	0.0023161	1.178311967	2.129977524
STX6	1.476460221	0.002357752	1.148568735	1.897957621
METAP2	1.652348271	0.002380513	1.195086902	2.28456592
R3HDM1	1.710383278	0.002400361	1.209471903	2.418750655
MYO19	1.437904915	0.002488834	1.136378593	1.819438134
RLF	1.600480578	0.00250877	1.179758321	2.17123968
DNM1L	1.526712087	0.00256467	1.159647317	2.009964375
METTL9	1.409403699	0.002601263	1.127283049	1.762129563
ARF3	1.479360265	0.002738488	1.144985931	1.911383131
RNF138	1.591209244	0.002763537	1.17387336	2.156916534
ETNK1	1.494355476	0.002772253	1.148607917	1.94417804
EXOSC3	1.57616851	0.002802784	1.16952969	2.124193336
PMS1	1.819256868	0.002831556	1.228209799	2.694731435
DUSP12	1.500067606	0.002859118	1.149154622	1.958137557
PRMT1	1.392462174	0.002912439	1.119735744	1.731614728
KPNA6	1.627095358	0.002985551	1.179941984	2.243702944
G2E3	1.770395861	0.002998884	1.214074054	2.581639477
PNRC2	1.552815199	0.00303694	1.160752122	2.077304016
UBAP2L	1.417079809	0.003129336	1.124527498	1.785741291
ABI2	1.472278884	0.003137842	1.139005438	1.903068273
DROSHA	1.506663701	0.003224454	1.146996466	1.979112906
NAA40	1.444788616	0.003228063	1.13098298	1.845663623
ACLY	1.374889603	0.003268398	1.112068784	1.69982419
MITD1	1.557063909	0.003342642	1.158395755	2.092935861
TDG	1.507329532	0.003351581	1.145887245	1.982780007
SUPT16H	1.524726078	0.00342669	1.149496844	2.022441058
PRKCI	1.43150385	0.003429369	1.125768774	1.82027013
TOP1	1.554129791	0.003430724	1.156729243	2.088059431
MEX3C	1.516759205	0.003432799	1.1474489	2.00493328
ZBTB40	1.6004713	0.003439275	1.167909582	2.193242031
CCDC93	1.518009509	0.003488817	1.147207635	2.008662425
CCAR1	1.586857734	0.003521631	1.16371188	2.163866771
VEZF1	1.464277197	0.00365433	1.13224206	1.893683148
MIS18A	1.393823123	0.003759623	1.113438251	1.744814224
MTPAP	1.768590451	0.003781356	1.202343357	2.601513258
SMPD4	1.584343212	0.003800586	1.160147304	2.163641983
ARL1	1.589938652	0.003821958	1.161246334	2.176889469
UIMC1	1.706886978	0.003887224	1.187331373	2.4537911
IFT52	1.4211476	0.00389311	1.119444537	1.804163078
SUZ12	1.448820257	0.003895562	1.126377203	1.863567667
STK4	1.564180197	0.004026826	1.153147313	2.121723444
UBE2O	1.543808076	0.004054387	1.148087459	2.075924928
IQCB1	1.533114833	0.004091794	1.145215174	2.052401282
XPO1	1.528383626	0.004179371	1.143317657	2.043138661
MRPL42	1.746481096	0.004192114	1.192344008	2.55815117
DCAF7	1.419060196	0.004210316	1.116620601	1.803416344

USP48	1.637758008	0.004339644	1.166891498	2.298629563
PSPC1	1.556344171	0.00436662	1.148183245	2.109599831
CBFB	1.404383446	0.004422822	1.111546258	1.774368678
DHX9	1.449926103	0.004430684	1.12259102	1.872708463
DNAJC10	1.424651793	0.004475878	1.116153571	1.818417091
HNRNPC	1.647697483	0.00459813	1.166495158	2.327405286
CWC22	1.64373195	0.004644885	1.165170935	2.318848369
HCFC1	1.527443778	0.004737779	1.138418094	2.049409182
KIF20B	1.497424204	0.004796519	1.131079386	1.982424287
THOC1	1.528900447	0.004804119	1.138219838	2.053677593
YEATS4	1.414503741	0.004834215	1.111352972	1.800346859
ADAM17	1.414154303	0.004883485	1.110959419	1.800094906
NCOA5	1.527483811	0.004894756	1.137183555	2.051741588
RAB5C	1.480120524	0.004918738	1.126193497	1.94527563
DHX15	1.471727723	0.004920165	1.1242442	1.92661211
NCBP1	1.675289492	0.004973536	1.168740525	2.401384072
RFC5	1.414421542	0.005091593	1.109736933	1.802759049
RFWD3	1.491271067	0.005108094	1.127392518	1.972595488
M6PR	1.345984958	0.005118346	1.093194039	1.657231419
BUB3	1.562312119	0.005303253	1.141693792	2.137892992
NOL7	1.434399599	0.00541545	1.11240432	1.849599262
ANKRD52	1.424419033	0.005518944	1.109499892	1.828724451
S100PBP	1.557787463	0.005534221	1.138938885	2.130669005
VHL	1.417763689	0.005658878	1.107170084	1.815487888
TCF3	1.349776829	0.00592276	1.09016141	1.671218107
LIN9	1.460643923	0.006152563	1.113841517	1.915425702
BCOR	1.425763716	0.006216432	1.10585855	1.838211744
NDE1	1.419963309	0.006377513	1.103723509	1.826812408
POLD3	1.440060805	0.006477942	1.107548187	1.872401713
ANKRD27	1.386858216	0.006534815	1.095657187	1.755453928
POLG2	1.428403685	0.006708188	1.103820726	1.848431579
PRPF40A	1.559203912	0.006833556	1.13011663	2.151208798
PGAM1	1.36983724	0.006951272	1.090005592	1.72150866
NAA25	1.684153349	0.007047044	1.152709362	2.460613748
PHF6	1.477838475	0.007181935	1.111619552	1.964706859
SART3	1.578920847	0.007280865	1.131082919	2.204074519
TBP	1.585932669	0.007290437	1.132372907	2.221160904
EWSR1	1.630943582	0.007385866	1.14028584	2.332728227
SUMO2	1.431092234	0.007456628	1.100630763	1.860773886
MTA2	1.511768233	0.007494758	1.116680318	2.046640522
STX12	1.56842078	0.007654755	1.126715905	2.183286604
FAM104A	1.557850226	0.007718833	1.124312496	2.158561198
HNRNPH3	1.532572113	0.007777401	1.119125475	2.09876134
CREB1	1.647867629	0.00843197	1.136387036	2.389562389
NRM	1.218070638	0.00846494	1.051714259	1.410740671
NPEPPS	1.358965932	0.008477046	1.081507749	1.707605338
E2F3	1.339950279	0.008602706	1.077174557	1.666829891
HEATR6	1.482533074	0.009050295	1.103054478	1.992561892
STRN4	1.439525656	0.009054419	1.094977376	1.892490348
CSNK2A1	1.420011185	0.009093954	1.091097307	1.848076932
SOCS7	1.501510179	0.009210964	1.105773709	2.038873596

PPP5C	1.429352071	0.009271569	1.092130493	1.870698928
COPB1	1.449669388	0.00929486	1.095838653	1.91774704
SMARCB1	1.353039477	0.009412361	1.076957751	1.699895679
COIL	1.525767737	0.009738082	1.107556649	2.101894461
HNRNPD	1.501998561	0.009951159	1.102373827	2.046492417
PUM1	1.52177829	0.010271654	1.104326089	2.097033826
HACE1	1.619300426	0.010458135	1.119651698	2.341919255
HNRNPA2B1	1.570251988	0.010505585	1.11136956	2.21860612
RBM8A	1.467034005	0.010764382	1.092760243	1.969497688
DCAF16	1.34368097	0.011213807	1.0694002	1.688309531
THUMPD2	1.52753332	0.011494302	1.099772447	2.121673487
ZNF248	1.477784435	0.011702572	1.09080092	2.002058116
ZBTB34	1.539043723	0.011747635	1.100507339	2.152330564
OTUD3	1.545078659	0.011928178	1.100667985	2.168926593
TMED2	1.410426502	0.01226017	1.077644001	1.845974102
DNAJC9	1.349978768	0.01227033	1.067399111	1.707367614
ACTR2	1.360707542	0.012617484	1.068216165	1.733286833
EHMT2	1.300660049	0.012875049	1.057305918	1.600025625
METTL3	1.493293727	0.013184535	1.087544907	2.050422138
ILF3	1.394520331	0.013260209	1.071846998	1.814332602
TTF1	1.562243487	0.014096996	1.09409377	2.230708902
USP24	1.564314533	0.014757837	1.091741107	2.241447117
DIABLO	1.618231857	0.015066787	1.097711561	2.385575989
DVL3	1.374223386	0.015237642	1.063046352	1.776488777
ZBED4	1.433234341	0.015812576	1.069940076	1.919883852
SOCS5	1.444161971	0.015965101	1.071003009	1.947337011
RNF219	1.447629503	0.015993976	1.071394256	1.95598508
MORF4L1	1.432773852	0.016032706	1.069221358	1.919940052
PHF21A	1.40424295	0.016137735	1.064934402	1.851661716
CHTOP	1.467601738	0.017052068	1.070841217	2.011367162
HNRNPA1	1.33758416	0.017375903	1.052531438	1.699836528
CDK11B	1.464287131	0.017611625	1.068729331	2.006248671
PPP4R1	1.400479527	0.01798402	1.059498284	1.851199699
TCERG1	1.417871836	0.018128357	1.061359791	1.894136711
TAF4	1.472817353	0.01827453	1.067831837	2.03139753
GTPBP2	1.268138506	0.018312499	1.04102171	1.544804738
PDCL	1.475649728	0.018680848	1.066981381	2.040843596
HMGXB4	1.43259494	0.018721176	1.061621403	1.933201662
FRMD8	1.323716708	0.018950472	1.047281211	1.67311884
FANCF	1.443193089	0.019416931	1.061032828	1.962998917
DEK	1.269340425	0.019810663	1.038595497	1.551349992
TUBB	1.239326995	0.019833215	1.034622652	1.484532933
EED	1.524998037	0.019998154	1.068724974	2.176068745
CHD4	1.419191394	0.020815279	1.054659964	1.909719038
PPP6R1	1.364259956	0.020864562	1.048243512	1.775546624
BRPF1	1.528375922	0.021452696	1.064710459	2.19396075
ZNF765	1.613377066	0.022080359	1.071173906	2.430030777
KCTD10	1.371285824	0.022158982	1.046264406	1.797274954
DPF2	1.461252539	0.022883916	1.053983035	2.025895022
WTAP	1.413959733	0.02292513	1.049092775	1.905724807
RIF1	1.441999124	0.02330163	1.051036448	1.978391404

PTBP2	1.44073222	0.023622159	1.050144464	1.97659408
ARMC8	1.592224315	0.02365823	1.06419673	2.382245874
TMEM39A	1.429833615	0.024086366	1.048001239	1.950784113
TJAP1	1.380684943	0.024144347	1.043085695	1.827549664
HAUS5	1.389499768	0.024239077	1.043754654	1.849773411
ZWILCH	1.341280306	0.024257936	1.038931114	1.731619004
USP36	1.376478877	0.024506744	1.041931547	1.818443933
ZCCHC3	1.347046972	0.025108438	1.03791381	1.748252626
APAF1	1.411683775	0.025837667	1.042442068	1.911713985
MAZ	1.268500021	0.025939988	1.028939517	1.563835655
ADO	1.437895251	0.025984838	1.044426304	1.979596591
SP3	1.368410569	0.026145995	1.037943578	1.804093714
LUC7L3	1.356354291	0.026682047	1.035861402	1.776006867
HNRNPA0	1.395680481	0.027138772	1.03837145	1.875941413
CDK2AP1	1.27964888	0.027284748	1.028028313	1.592856185
ANAPC5	1.469867261	0.027450378	1.043741043	2.06996724
DIP2B	1.390592704	0.027459449	1.037311134	1.864192917
CSTF3	1.425076962	0.027936011	1.039134211	1.954361935
SBNO1	1.501560546	0.029461944	1.041402621	2.165045515
ADNP	1.335569693	0.029939073	1.028513948	1.734294812
RNF41	1.506698197	0.030361628	1.039645522	2.183570659
MIS18BP1	1.354271333	0.030378931	1.029151862	1.782099331
EFCAB7	1.498742643	0.030446053	1.03892986	2.162060785
LEMD3	1.45272811	0.030890143	1.034943118	2.039164206
HP1BP3	1.317251556	0.031688669	1.024452581	1.693735458
RPS6KB1	1.428089599	0.032507561	1.030135463	1.979778365
ZNF224	0.712662687	0.032635928	0.522307385	0.972393116
PGS1	1.376919398	0.032793624	1.02651298	1.846939168
EIF4H	1.435937328	0.034165187	1.027383962	2.00695756
CNOT3	1.406224992	0.034184382	1.025746626	1.92783352
AZI2	1.436591052	0.034330787	1.027101264	2.009338245
SLMAP	1.47463412	0.035583003	1.026533011	2.118339853
RYK	1.439110456	0.03568718	1.024650144	2.021215648
UBA3	1.475398451	0.035703663	1.026325237	2.120965667
ZBTB12	1.244815887	0.036450834	1.013903611	1.528317462
FUS	1.31803184	0.037791175	1.015696102	1.710361916
HGS	1.251134015	0.037945828	1.012543967	1.545944052
HMGB1	1.413586385	0.03870808	1.018130078	1.962643586
BAZ2A	1.327649134	0.03876344	1.014743109	1.737042813
METTL4	1.507836423	0.040240759	1.018447881	2.232387852
PAPOLG	1.631932503	0.040253735	1.022008433	2.605852954
EP400	1.426767014	0.040286927	1.015865909	2.003870879
ERCC3	1.423010419	0.0403779	1.015591455	1.993871299
C18orf25	1.510430603	0.041048495	1.016910544	2.243462437
SENP5	1.502562879	0.041356099	1.016091712	2.221940382
RERE	1.29339507	0.042345136	1.008929323	1.658065405
ZNF202	1.445277742	0.042566876	1.012421012	2.063200711
U2SURP	1.360519398	0.042710773	1.010163492	1.832389556
NUP160	1.347293563	0.043560417	1.008652765	1.799628186
SUDS3	1.399929991	0.044625487	1.008108096	1.944041505
PDE7A	1.247223289	0.044811342	1.005128326	1.547629186

ZNF26	1.393741087	0.044908672	1.007567988	1.927923716
CDK11A	1.383652563	0.04492318	1.007380116	1.900468735
RUNDC1	1.360053229	0.045200981	1.00659589	1.837624021
PHACTR4	1.35835722	0.047776327	1.003004603	1.839607048
SCLT1	1.549485592	0.048537512	1.002816234	2.394163076
TBK1	1.455753193	0.049901345	1.000161785	2.118874557

Table S7. Importance of SRSF-related genes when OBB error rate reached lowest.

Signature	Importance
UTP6	0.699528597
SOCS7	0.563189568
PGAM5	0.548090708
CCDC59	0.478713426
SF3B2	0.47092414
PGAM1	0.457258419
TUBA1C	0.456262637
LLPH	0.455818072
NUP43	0.441228558
FAM72A	0.438426616
PRMT1	0.437179298
MAPRE1	0.434113355
ANKRD27	0.422022243
PLIN3	0.417791508
WDR75	0.41551843
DDX54	0.411236857
CORO1C	0.3954568
DDX11	0.395314381
GTPBP4	0.384773425
SNRPB2	0.373684259
ETNK1	0.372485793
PSMD9	0.371797822
QSER1	0.37080448
CCDC93	0.365922823
GRPEL2	0.364913119
XPO5	0.359760311
NAA25	0.356107581
ZNF131	0.355668566
FOXK2	0.355208033
NIF3L1	0.354090849
MIS18A	0.352835699
NAP1L1	0.352639988
PPP5C	0.351610152
GTF3C2	0.351502379

Table S8. Coefficients of members involved in SRSF-related signature.

id	coef	exp(coef)	se(coef)	z	Pr(>|z|)
SRSF1	−2.353467349	0.095039057	0.516754866	−4.554320638	5.26E-06
SRSF2	0.948122406	2.580859301	0.424673535	2.232591221	0.025575914
SRSF11	−0.439922027	0.64408664	0.290621643	−1.513727685	0.13009492
WDR75	0.758899371	2.135924067	0.383289155	1.979965674	0.047707386

TUBA1C	0.429508257	1.536501774	0.160957461	2.668458206	0.007620026
SNRPB2	0.381780958	1.464891177	0.259623162	1.470519638	0.141421071
PGAM5	0.431431385	1.539459507	0.273125994	1.57960573	0.114197186
NUP43	0.527707751	1.695042393	0.268794843	1.963236146	0.049618741
NAA25	−0.936032758	0.392180629	0.537500338	−1.741455199	0.08160382
MIS18A	−0.563503149	0.569211535	0.218822983	−2.575155224	0.010019511
MAPRE1	−0.428468931	0.651505832	0.241418531	−1.774797192	0.07593138
GTPBP4	0.502819434	1.65337629	0.267823703	1.877426936	0.060459603
GTF3C2	0.555001875	1.741944251	0.336659238	1.648556798	0.099238466
GRPEL2	1.095717432	2.991327987	0.272274147	4.024316829	5.71E-05
FAM72A	0.840040439	2.31646065	0.261605707	3.21109371	0.001322308
ETNK1	0.554378653	1.740858972	0.260563533	2.127614125	0.03336909
DDX54	−0.551057921	0.576339766	0.331388832	−1.662874147	0.096337631
ANKRD27	−0.338259333	0.713010357	0.230986109	−1.464414174	0.143080818
